# Post‐Discharge Opioid Prescribing After Elective Colorectal Resection: An International Survey

**DOI:** 10.1002/wjs.70245

**Published:** 2026-01-29

**Authors:** Ghadeer Olleik, Hiba Elhaj, Samin Shirzadi, Francesca Fermi, Maxime Lapointe‐Gagner, Sender Liberman, Mohsen Alhashemi, Tahereh Najafi Ghezeljeh, Fatemeh Rajabiyazdi, Nawar Touma, Pepa Kaneva, Agnihotram V. Ramanakumar, Badma Bashankaev, Alexandra Sidorova, Stephen J. Chapman, Chuan‐Gang Fu, Lucia Oliveira, Sofia Valanci, Audrius Dulskas, Steven Wexner, Lawrence Lee, Liane S. Feldman, Marylise Boutros, Julio F. Fiore

**Affiliations:** ^1^ Department of Surgery McGill University Montreal Quebec Canada; ^2^ Centre for Outcomes Research and Evaluation (CORE) Research Institute of the McGill University Health Centre Montreal Quebec Canada; ^3^ Perioperative Care and Outcomes Research (PCOR) Lab McGill University Montreal Quebec Canada; ^4^ Surgery Department, Faculty of Medicine Kuwait University Health Sciences Center Jabriyah Kuwait; ^5^ Department of Computer Science University of Calgary Calgary Alberta Canada; ^6^ Department of Community Health University of Calgary Calgary Alberta Canada; ^7^ Global Medical System Hospital Moscow Russia; ^8^ Leeds Institute of Medical Research University of Leeds Leeds UK; ^9^ Department of Colorectal Surgery Shanghai East Hospital Tongji University Shanghai China; ^10^ Clinica de Coloproctologia Rio de Janeiro Brazil; ^11^ Centro Medico ABC Ciudad de México Mexico; ^12^ Faculty of Medicine Institute of Clinical Medicine Vilnius University Vilnius Lithuania; ^13^ Ellen Leifer Shulman and Steven Shulman Digestive Disease Center Cleveland Clinic Weston Florida USA

**Keywords:** colorectal, international, opioids, postoperative analgesia, surgery, survey

## Abstract

**Background:**

Excessive opioid prescribing after colorectal surgery can lead to adverse events and contribute to the opioid crisis. Understanding international prescribing patterns is essential for guiding practice and future research. The Analgesia After Colorectal Surgery (ACORE) survey aimed to characterize international opioid prescribing practices after elective colorectal resection.

**Method:**

This international cross‐sectional survey followed established methodological guidelines. Eligible participants were colorectal, gastrointestinal, and general surgeons, as well as surgery trainees. Recruitment followed snowball sampling via international surgical societies' mailing lists, social media, and personal networks. The primary outcome of interest was post‐discharge opioid prescribing after open and MIS elective colorectal resection. Secondary outcomes included prescription quantity in morphine milligram equivalents (MMEs). Data were analyzed using descriptive statistics and logistic regression with Bayesian model averaging.

**Results:**

Among 817 participants, 88% were surgeons, 12% were trainees, 62% practiced in academic hospitals, and 67% had over 5 years in practice. Overall, 57% of the participants reported prescribing opioids at discharge (55% after open and 54% after minimally invasive procedures). Opioids were commonly prescribed by surgeons practicing in Australia and New Zealand (100%), Northern America (92%), Northern Europe (68%), and South‐eastern Asia (71%). In contrast, they were less frequently prescribed in Eastern Europe (11%), Eastern Asia (22%), Latin America and the Caribbean (26%), Southern Europe (19%), and Northern Africa (0%). The median quantity of opioids prescribed at discharge varied widely (30–200 MMEs). In regression analysis accounting for surgeon and practice characteristics, region of practice was the only factor independently associated with opioid prescribing.

**Conclusion:**

The extensive global variation in opioid prescribing underscores clinical equipoise and challenges the assumption that post‐discharge opioids are universally necessary for patients undergoing colorectal resection.

## Graphical abstract

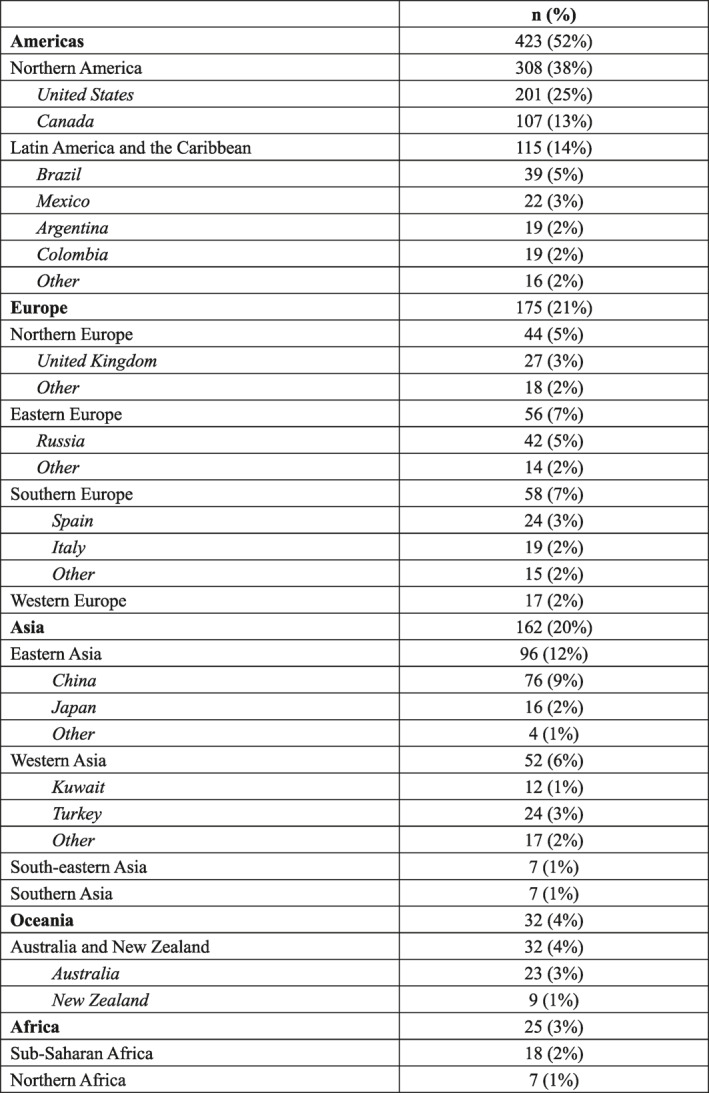

## 1 Introduction

Postoperative opioid prescribing can lead to adverse events and contribute to the opioid crisis, which originated in North America but now poses a global threat, with rising opioid‐related morbidity and mortality worldwide [1, 2]. Patients undergoing colorectal resection are particularly vulnerable to opioid‐related harms. Compared with other procedures, they have among the highest rates of new persistent opioid use, with approximately 10% continuing opioid therapy beyond 3 months after surgery [3, 4]. Moreover, opioid use may lead to postoperative ileus, prolonging hospital stay and increasing healthcare costs [5]. While opioid‐sparing strategies including non‐steroidal anti‐inflammatory drugs (NSAIDs) are recommended [6, 7], they are often overlooked due to concerns about anastomotic leakage [8, 9, 10]. Therefore, colorectal surgeons face the complex challenge of ensuring adequate postoperative pain relief while minimizing harm.

Pain management after colorectal surgery is continually evolving, with no clear consensus on the safest and most effective approach [6, 7]. Analgesic choices often depend on surgeons' preferences and local care culture [11], yet international prescribing variations remain poorly understood. This knowledge is essential for guiding practice and future research, particularly in settings where certain analgesic approaches remain over‐ or under‐utilized. The Analgesia After Colorectal Surgery (ACORE) survey aimed to assess international patterns of analgesia prescribing after elective colorectal resection.

## 2 Material and Methods

The ACORE survey followed best‐practice survey guidelines [12, 13, 14] and was approved by McGill University's ethics board (A04‐E29‐19B). Informed consent was implied through participation. Reporting adhered to the Consensus‐Based Checklist for Reporting of Survey Studies (CROSS) [15] (Supporting Information S1: Table S1) and the Checklist for Reporting Results of Internet E‐Surveys (CHERRIES) [16] (Supporting Information S1: Table S2).

### 2.1 Survey Design

A web‐based survey (LimeSurvey v.5) was designed by a team of colorectal surgeons, general surgeons, anesthetists, pharmacists, and scientists representing eight countries (Brazil, Canada, China, Kuwait, Mexico, Russia, United Kingdom [U.K.], and the United States [U.S.]). The survey content was informed by previous literature [11, 17], the team's clinical and methodological expertise, and pilot testing. The final version of the survey (Supporting Information S1: Table S3) contained 31 questions eliciting participants' characteristics and analgesia prescribing practices in‐hospital and post‐discharge. Participants could report the use of different analgesia approaches for open and minimally invasive surgery (MIS; i.e., laparoscopic, robotic).

The survey was designed in English and pilot tested by ten native‐speaking surgeons and five trainees, with revisions based on their feedback. Bilingual team members translated it into French, Arabic, Spanish, Portuguese, Russian, and Mandarin. These languages were chosen because they are spoken in over 75% of the global land area [18]. Translations were pilot‐tested by at least five native‐speaker surgeons or trainees to ensure conceptual and linguistic equivalence with the original survey.

To facilitate participation, the survey required no access password and could be completed in approximately 5 min [19, 20]. Adaptive questions (i.e., displayed based on previous responses) reduced response burden. Most questions were multiple‐choice, with open‐text fields used for drug dosages and comments. All questions (except text fields) were mandatory, presented in fixed order, and could be reviewed before submission. To ensure data integrity, the two “captcha” questions assessing colorectal surgery technical knowledge were used to validate responses (Supporting Information S1: Table S3), and a digital cookie prevented multiple submissions from the same IP address.

### 2.2 Survey Validity

Because the survey aimed to capture descriptive data on prescribing practices rather than quantifying latent constructs (e.g., pain, quality of life), formal psychometric testing (e.g., reliability, construct validity) was not appropriate [13]. Following guidelines for survey research [12, 13, 14], face and content validity were established through literature review, expert input, and pilot testing to confirm item relevance, clarity, and flow. For translated versions, native‐speaking clinicians confirmed conceptual equivalence and linguistic accuracy, supporting the survey's robustness and cross‐cultural relevance.

### 2.3 Participants and Eligibility Criteria

Colorectal, gastrointestinal, general surgeons, and surgical trainees (residents, registrars, and fellows) were eligible to participate. We excluded those who (1) indicated not performing colorectal resections, (2) exited the survey prior to completing all the questions, and (3) did not answer both “captcha” questions correctly. Participation was voluntary and not compensated. Responses were automatically anonymized in LimeSurvey, with no identifiable personal information collected.

### 2.4 Survey Dissemination

To reach a large, geographically dispersed participant sample, we employed non‐probabilistic snowball sampling [12, 21, 22, 23] using the following approaches:

1. Contacting colorectal/proctology and general surgery societies identified through a targeted Google search and inviting them to distribute the survey via email or social media.
2. Creating an X (formerly Twitter) account to disseminate the survey (Supporting Information S1: Figure S1) [24, 25]. Survey‐related posts were shared via team members' personal and institutional accounts. Partner surgical societies, colleagues, and survey participants were encouraged to repost. X accounts of self‐identified colorectal, gastrointestinal, and general surgeons were followed to raise survey awareness.
3. Inviting colleagues within team members' professional networks to complete and circulate the survey via email and social media.

### 2.5 Data Analysis

Given the exploratory and descriptive nature of the study and the use of snowball sampling, no formal power calculation was performed [21]. This approach aligns with recommendations for international surveys where the objective is to capture diverse, real‐world practices and probabilistic sampling is not feasible [21].

Responses were analyzed by continental region, subregion, and country according to United Nations (U.N.) codes [26]. Participation (survey initiations/visitors) and completion rates (completed/initiated surveys) were calculated according to CHERRIES recommendations [16]. The primary analysis focused on the rate of surgeons who prescribed opioids at discharge (yes/no), analyzed overall and stratified by open versus MIS. Opioid quantities were calculated in morphine milligram equivalents (MME) [27]. For countries where opioid pills are pre‐packed (i.e., prescribers do not determine the number of pills dispensed at the pharmacy), a 3‐day opioid supply was assumed (∼4 pills/day; standard pack of 14 pills), with sensitivity analysis considering a 6‐day supply (28 pills). Missing data were minimal (*n* = 4 open‐text fields, < 1%) and therefore not imputed.

Factors associated with opioid prescribing were evaluated using multivariable logistic regression with Bayesian model averaging (BMA), which integrates multiple models into the predictive process to account for model uncertainty and identify the most probable predictors across competing models [28, 29, 30]. Potential predictors included surgeons' subregion of practice, years of experience, number of annual resections, practice setting (academic vs. non‐academic), length of stay, and NSAID prescribing. Odds ratios (OR) were derived from averaged coefficients, with OR = 1 indicating no association [28, 29, 30]. To assess the robustness of observed associations, posterior effect probabilities (PEP) were calculated and classified as: “no evidence” of association (PEP < 50%), “weak evidence” (50%–75%), “positive evidence” (75%–95%), “strong evidence” (95%–99%), and “very strong evidence” (> 99%) [30, 31]. All statistical analyses were conducted by G.O. and F.F (graduate trainees), under supervision of J.F. (surgical outcomes researcher) and A.V.R. (biostatistician), using Stata 18 (StataCorp) and R 3.4.0 (R Foundation).

## 3 Results

Among 29 surgical societies contacted, 13 shared the survey via email (*n* = 10), X (*n* = 1), or both (*n* = 2) (participating societies listed in the Acknowledgment section). Our X account followed 2691 potential participants' accounts and was followed back by 589. We received 1225 survey‐site visits and 1025 responses from October 21, 2021, until April 8, 2022. After excluding 208 responses (107 incomplete, 39 non‐colorectal surgeons, and 62 incorrect “captcha”), 817 were included in the analysis (Supporting Information S1: Figure S2), corresponding to a participation rate of 84% and a completion rate of 80%.

Participants represented 67 countries across 5 continental regions and 13 subregions (Table 1 and Supporting Information S1: Table S4). Most respondents were from the Americas (*n* = 423 [52%]), Europe (*n* = 175 [21%]) and Asia (*n* = 162 [20%]). The most represented subregions were Northern America (*n* = 308 [38%]), Latin America/Caribbean (*n* = 115 [14%]), Eastern Asia (*n* = 96 [12%]), and Southern Europe (*n* = 58 [7%]). The countries most represented were the United States (*n* = 201 [25%]), Canada (*n* = 107 [13%]), China (*n* = 79 [9%]), Russia (*n* = 42 [5%]), and Brazil (*n* = 39 [5%]).

**TABLE 1:** Survey participants' continental region, subregion, country of practice, and participation rates (*n* = 817).

	*n* (%)
Americas	423 (52)
Northern America	308 (38)
United States	201 (25)
Canada	107 (13)
Latin America and the Caribbean	115 (14)
Brazil	39 (5)
Mexico	22 (3)
Argentina	19 (2)
Colombia	19 (2)
Other	16 (2)
Europe	175 (21)
Northern Europe	44 (5)
United Kingdom	27 (3)
Other	18 (2)
Eastern Europe	56 (7)
Russia	42 (5)
Other	14 (2)
Southern Europe	58 (7)
Spain	24 (3)
Italy	19 (2)
Other	15 (2)
Western Europe	17 (2)
Asia	162 (20)
Eastern Asia	96 (12)
China	76 (9)
Japan	16 (2)
Other	4 (1)
Western Asia	52 (6)
Kuwait	12 (1)
Turkey	24 (3)
Other	17 (2)
South‐eastern Asia	7 (1)
Southern Asia	7 (1)
Oceania	32 (4)
Australia and New Zealand	32 (4)
Australia	23 (3)
New Zealand	9 (1)
Africa	25 (3)
Sub‐Saharan Africa	18 (2)
Northern Africa	7 (1)

*Note:* Data are reported as *n* (%). A complete list of the represented countries is available in Supporting Information S1: Table S4.

### 3.1 Participants' Demographic and Practice Characteristics

Overall, 88% of the participants were practicing surgeons (colorectal *n* = 486 [59%], general *n* = 152 [19%], gastrointestinal *n* = 85 [10%]), and 12% were trainees (senior *n* = 73 [9%], junior *n* = 21 [3%]) (Table 2). Most practiced in academic (*n* = 507, [62%]) or community hospitals (*n* = 214 [26%]), performed more than 30 colorectal resections annually (*n* = 488 [60%]), and had more than 5 years in practice (*n* = 544 [67%]). The majority used both open and MIS approaches (*n* = 583 [71%]) and prescribed the same analgesia regimen for both approaches in‐hospital (*n* = 353 [61%]) and post‐discharge (*n* = 445 [76%]). Use of enhanced recovery pathways varied by region (e.g., < 50% in Sub‐Saharan Africa and Latin America/Caribbean, > 90% in Northern America and Northern Europe). The typical length of stay was widely variable (e.g., median of 5 days after open surgery and 3 after MIS in Northern America; median of 10 days after open and 7 after MIS in Eastern Asia; Supporting Information S1: Table S5).

**TABLE 2:** Survey participants' demographic and practice characteristics (*n* = 817).

Current position	
Practicing colorectal surgeon/proctologist	486 (59%)
Practicing gastrointestinal surgeon	85 (10%)
Practicing general surgeon	152 (19%)
Trainee (resident/registrar/fellow)	94 (12%)
Junior (≤ 3 years of training)	21 (3%)
Senior (> 4 years of training)	73 (9%)
Predominant practice/training location	
Academic hospital	507 (62%)
Community hospital	214 (26%)
Other (including private practice)	96 (12%)
Years of independent practice	
None	94 (11%)
≤ 5 years	179 (22%)
6–10 years	153 (19%)
11–20 years	213 (26%)
≥ 21 years	178 (22%)
Procedures performed annually	
1–10	95 (11%)
11–30	234 (29%)
31–60	219 (27%)
61–100	154 (19%)
> 100	115 (14%)
Surgical approach used in practice	
Both open and minimally invasive	583 (71%)
Only minimally invasive (unless converted)	177 (22%)
Only open	57 (7%)
Use of enhanced recovery pathways	
Yes	639 (78%)
No	178 (22%)
Length of hospital stay, median (IQR)	
Open surgery	5 (5–7)
Minimally invasive surgery	4 (3–5)

*Note:* Data are reported as *n* (%), unless otherwise stated.

Abbreviation: IQR, Interquartile range.

### 3.2 In‐Hospital Analgesia

Peripheral nerve blocks were commonly used in Northern America, Northern Europe, Australia/New Zealand (> 50%) but were less frequent elsewhere (< 20% in Latin America/Caribbean, Eastern and Western Europe, and Western Asia) (Supporting Information S1: Table S5). Epidural analgesia was frequently used in many regions (> 60% in Eastern, Southern, and Western Europe), particularly after open procedures. In Eastern Europe, many surgeons (> 50%) reported using epidural analgesia after MIS. There were wide variations in the use of in‐hospital spinal analgesia (0%–50%), wound infiltration (7%–100%), intravenous (64%–100%), and oral analgesia (40%–100%).

In‐hospital opioid prescribing was common in most regions (> 80% in Northern America, Northern and Western Europe, Western, South‐eastern, and Southern Asia, Australia/New Zealand, and Sub‐Saharan and Northern Africa) but less common in Eastern (39%) and Southern Europe (45%). In‐hospital NSAID use was common in Northern America, Eastern Europe, South‐eastern and Southern Asia, Sub‐Saharan and Northern Africa (≥ 70%) but less common elsewhere (41% in Western Europe, 48% in Australia/New Zealand). Gabapentinoids were predominantly prescribed in Northern America (48%) but rarely elsewhere (0%–14%). Dipyrone/metamizole was predominantly prescribed in Latin America/Caribbean, and Southern and Western Europe (> 40%) but not elsewhere (0%–21%). There were regional variations in the use of traditional local anesthetics (19%–86%), sustained release local anesthetics (0%–29%), and intravenous acetaminophen/paracetamol (27%–86%; Supporting Information S1: Table S5).

### 3.3 Post‐Discharge Analgesia

Overall, 57% of the participants reported prescribing opioids at discharge (55% after open and 54% after MIS procedures). Prescription rates were higher in Australia/New Zealand (100%), Northern America (92%), Northern Europe (68%), and South‐eastern Asia (71%), but markedly lower in Eastern Europe (11%), Eastern Asia (22%), Latin America/Caribbean (26%), Southern Europe (19%), and Northern Africa (0%; Table 3 and Supporting Information S1: Table S4). Countries with the highest rates of opioid prescribing were Australia (100%), the U.S. (93%), Canada (90%), and the U.K. (78%). The lowest rates were observed in Japan (0%), Spain (8%), Russia (14%), Italy (16%), and Colombia (16%; Supporting Information S1: Table S4). Oxycodone was most commonly prescribed in Northern America (46%) and Australia/New Zealand (47%), codeine in Northern Europe (50%), and tramadol in all other subregions (> 60%; Supporting Information S1: Table S6).

**TABLE 3:** Post‐discharge opioid prescribing practices by continental region, subregion, and country of practice.

	All (%)	Open (%)	MIS (%)
Americas (*n* = 423)	313 (74)	219 (73)	288 (71)
Northern America (*n* = 308)	283 (92)	195 (92)	270 (89)
United States (*n* = 201)	187 (93)	144 (94)	180 (90)
Canada (*n* = 107)	96 (90)	51 (88)	90 (87)
Latin America and the Caribbean (*n* = 115)	30 (26)	24 (27)	18 (18)
Brazil (*n* = 39)	15 (38)	10 (37)	8 (25)
Mexico (*n* = 22)	5 (23)	5 (24)	3 (16)
Argentina (*n* = 19)	4 (21)	3 (21)	3 (18)
Colombia (*n* = 19)	3 (16)	3 (23)	1 (6)
Other (*n* = 16)	3 (19)	3 (21)	3 (21)
Europe (*n* = 175)	56 (32)	48 (33)	46 (28)
Northern Europe (*n* = 44)	30 (68)	28 (68)	25 (64)
United Kingdom (*n* = 27)	21 (78)	19 (79)	18 (72)
Other (*n* = 18)	9 (53)	9 (53)	7 (50)
Eastern Europe (*n* = 56)	6 (11)	3 (7)	4 (8)
Russia (*n* = 42)	6 (14)	3 (9)	4 (11)
Other (*n* = 14)	0 (0)	0 (0)	0 (0)
Southern Europe (*n* = 58)	11 (19)	10 (20)	8 (14)
Spain (*n* = 24)	2 (8)	2 (10)	2 (8)
Italy (*n* = 19)	3 (16)	2 (13)	3 (16)
Other (*n* = 15)	6 (40)	6 (40)	3 (23)
Western Europe (*n* = 17)	9 (53)	7 (64)	9 (53)
Asia (*n* = 162)	54 (33)	48 (34)	44 (29)
Eastern Asia (*n* = 96)	21 (22)	17 (21)	19 (20)
China (*n* = 76)	19 (25)	15 (23)	17 (23)
Japan (*n* = 16)	0 (0)	0 (0)	0 (0)
Other (*n* = 4)	2 (50)	2 (50)	2 (50)
Western Asia (*n* = 52)	24 (46)	22 (47)	20 (43)
Kuwait (*n* = 12)	6 (50)	4 (40)	5 (50)
Turkey (*n* = 24)	8 (33)	8 (33)	7 (32)
Other (*n* = 17)	10 (63)	10 (77)	8 (57)
South‐eastern Asia (*n* = 7)	5 (71)	5 (71)	3 (43)
Southern Asia (*n* = 7)	4 (57)	4 (57)	2 (40)
Oceania (*n* = 32)	32 (100)	27 (100)	28 (90)
Australia and New Zealand (*n* = 32)	32 (100)	27 (100)	28 (90)
Australia (*n* = 23)	23 (100)	18 (100)	21 (91)
New Zealand (*n* = 9)	9 (100)	9 (100)	7 (88)
Africa (*n* = 25)	11 (44)	11 (44)	3 (23)
Sub‐Saharan Africa (*n* = 18)	11 (61)	11 (61)	3 (50)
Northern Africa (*n* = 7)	0 (0)	0 (0)	0 (0)

*Note:* Data are reported as *n* (%). Countries represented in ≥ 10 participants are listed in the table. A complete list of the represented countries and their rates of opioid prescribing is available in Supporting Information S1: Table S4.

Abbreviation: MIS, minimally invasive surgery.

The median quantity of opioids prescribed post‐discharge varied widely, ranging from 30 to 200 MMEs. The highest amounts were reported in Australia/New Zealand (median 186.4 MMEs [89–560] after open, 200 MMEs [92.5–518.75] after MIS), Northern Europe (median 140 MMEs [70–262.5] after open, 129.5 MMEs [63–213.5] after MIS), and Northern America (median 120 MMEs [75–162.5] after open, 90 MMEs [60–150] after MIS; Table 4). Notably, wide IQRs indicate substantial variability in prescribing practices within regions. Results were consistent in sensitivity analyses (Supporting Information S1: Table S7).

**TABLE 4:** Total Morphine Milligram Equivalents (MME) prescribed in different subregions.

	Open	MIS
All regions	105 MMEs [63–162.5]	90 MMEs [60–150]
Americas		
Northern America	120 MMEs [75–162.5]	90 MMEs [60–150]
Latin America and the Caribbean	50 MMEs [21.25–77.5]	63 MMEs [30–140]
Europe		
Northern Europe	140 MMEs [70–262.5]	129.5 MMEs [63–213.5]
Eastern Europe	86.4 MMEs [5–90]	30 MMEs [15–55]
Southern Europe	90 MMEs [63–200]	86.4 MMEs [42–100]
Western Europe	120 MMEs [18–191.4]	70 MMEs [37.5–150]
Asia		
Eastern Asia	86.4 MMEs [30–210]	86.4 MMEs [30–210]
Western Asia	40 MMEs [9–70]	60 MMEs [25–107.5]
South‐eastern Asia	75 MMEs [75–75]	45 MMEs [25–75]
Southern Asia	72.5 MMEs [60–87.5]	85 MMEs [70–100]
Oceania		
Australia and New Zealand	186.4 MMEs [89–560]	200 MMEs [92.5–518.75]
Africa		
Sub‐Saharan Africa	70 MMEs [50–136.4]	100 MMEs [100–200]
Northern Africa	—	—

*Note:* Data are reported as median MME [IQR]. For countries where opioid pills are pre‐packed (i.e., the prescriber does not determine the number of pills received by patients at the pharmacy), this analysis considered that patients receive a standard box of 14 pills (3‐day supply, ∼4 pills per day).

Post‐discharge acetaminophen/paracetamol was prescribed in most regions (> 70% in Northern America, Northern and Southern Europe, Western, South‐eastern and Southern Asia, Northern Africa, Australia/New Zealand). NSAIDs prescribing was common in Latin America/Caribbean, Eastern Europe, Western Asia, South‐Eastern Asia, Sub‐Saharan, and Northern Africa (≥ 60%) but not elsewhere (< 50% in Western Europe, Australia/New Zealand; Supporting Information S1: Table S8). Types of NSAIDs prescribed varied across different subregions (Supporting Information S1: Table S9). Gabapentinoids were predominantly prescribed in Northern America (19%) but rarely elsewhere (0%–7%). Dipyrone/metamizole use was common in Latin America/Caribbean and in Southern and Western Europe (> 30%) but rare elsewhere (0%–6%; Supporting Information S1: Table S8). Non‐pharmacological pain interventions were used by 144 participants (18%), most commonly cold therapy (53%), physical activity (45%), localized heat (42%), and relaxation techniques (33%).

### 3.4 Factors Associated With Post‐Discharge Opioid Prescribing

In BMA analysis focused on overall opioid prescribing (regardless of surgical approach), subregion of practice was the only factor independently associated with opioid prescribing (PEP = 100%, supporting very strong evidence of association; Table 5). Similar results were observed in subgroup analyses focused on open and MIS procedures, with region of practice remaining the sole factor associated with opioid prescribing (PEP = 100%; Supporting Information S1: Tables S10 and S11).

**TABLE 5:** Bayesian model averaging (BMA) analysis of potential predictors of opioid prescribing at discharge after both open and minimally invasive surgery (multiple BMA analysis; *n* = 817).

Potential predictor	OR [95% CI]	PEP
Subregion of practice		100
Northern America	Reference	
Latin America and the Caribbean	0.03 [0.02–0.06]	
Western Asia	0.08 [0.04–0.15]	
South‐eastern Asia	0.22 [0.04–1.21]	
Southern Asia	0.12 [0.02–0.56]	
Eastern Asia	0.02 [0.01–0.05]	
Northern Europe	0.19 [0.09–0.40]	
Eastern Europe	0.01 [0.00–0.03]	
Western Europe	0.10 [0.03–0.28]	
Southern Europe	0.02 [0.01–0.04]	
Sub‐Saharan Africa	0.14 [0.05–0.39]	
Australia and New Zealand^a^	100% opioid prescription rate	
Northern Africa^a^	0% opioid prescription rate	
Years of independent practice		0
0	Reference	
1–6	1	
6–10	1	
11–20	1	
> 20	1	
Practice setting		0
Academic	Reference	
Community or other	1	
Average length of stay, days	1	0
Procedures performed annually		0
< 11	Reference	
11–30	1	
31–60	1	
61–100	1	
> 100	1	
NSAIDs prescription at discharge (yes)	0.98 [0.82–1.17]	8

*Note:* In Bayesian model averaging, ORs are calculated from the mean of the beta coefficients (weighted averages) of all possible models and PEP (reported in %) reflects how likely it is for specific variables to predict the outcome of interest. ORs should be interpreted the between‐group difference in odds of opioid prescribing (for nominal/dichotomous predictors) or difference in odds for every 1‐unit change (for continuous predictors), assuming all other variables are held constant. When the OR is exactly 1, it means there is no association between the predictor and the outcome (PEP = 0).

Abbreviations: CI, Confidence Interval; NSAIDs, non‐steroidal anti‐inflammatory drugs; OR, odds ratio; PEP, posterior effect probability.

^a^ Variables indicating perfect prediction were omitted from the model.

## 4 Discussion

The ACORE survey revealed substantial regional differences in post‐discharge opioid prescribing after elective colorectal resection, with higher prescription rates in Australia/New Zealand, Northern America, Northern Europe, and South‐eastern Asia. In contrast, prescription rates were lower in Eastern Europe, Eastern Asia, Latin America/Caribbean, Southern Europe, and Northern Africa. The quantity of opioids prescribed varied widely across and within regions, suggesting limited standardization. While acetaminophen/paracetamol was commonly prescribed globally, NSAID prescribing was considerably variable. Dipyrone/metamizole, an atypical NSAID that targets COX‐3 enzymes in the central nervous system [32], was frequently prescribed in several regions. Non‐pharmacological pain interventions were rarely prescribed.

Previous cohort studies have revealed international disparities in postoperative pain management, typically focusing on head‐to‐head comparisons between the U.S. and other countries [23, 33, 34, 35]. However, this literature has not focused specifically on colorectal surgery and has not examined opioid prescribing across diverse international settings. The main strength of our study is its global scope: broad dissemination of our multilingual survey through surgical societies, social media, and professional networks enabled participation from more than 800 surgeons representing 67 countries across five continents. The participation of surgeons from academic and non‐academic settings, with varying levels of experience and case volumes, contributes to a comprehensive overview of prescribing practices. The study adhered to best‐practice guidelines for the conduct [12, 13, 14] and reporting [15, 16] of survey research. Given these strengths, this study contributes novel, colorectal resection–specific insights into global variations in analgesia prescribing, offering valuable knowledge to inform prescribing practices and future research.

Our survey revealed that pain management discrepancies emerge early during hospital stay. While multimodal analgesia was common, specific modalities varied widely. Current guidelines recommend the use of peripheral nerve blocks and local wound infiltration after open and MIS colorectal procedures; [6, 7] however, their use was largely limited to Northern America, Northern Europe, and Australia/New Zealand. Conversely, despite guidelines discouraging epidural analgesia after MIS procedures [6, 7], this practice remains common in Eastern, Southern, and Western Europe. These findings highlight the need for better translation of evidence into routine care to ensure optimal in‐hospital analgesia practices globally.

Northern America has long been recognized as an outlier in postoperative opioid prescribing [23, 33, 34, 35]. However, in colorectal surgery, similar patterns were observed in Australia/New Zealand, South‐eastern Asia, and Northern Europe. Since the early 2000s, these regions have experienced sharp increases in opioid prescribing [2, 36, 37, 38]. The higher prescription rates in these regions may reflect shared cultural perceptions of pain (i.e., fatalistic, requiring prompt intervention) and beliefs about opioid efficacy and safety [39]. In contrast, the lower prescription rates in Latin America/Caribbean, Eastern Asia, and Southern and Eastern Europe may reflect different cultural attitudes toward pain, stricter opioid prescribing regulations, and greater reliance on non‐opioid strategies. Variations in prescribing may also reflect differences in individual surgeon, hospital, and care characteristics (i.e., length of stay); however, in our regression analysis, region of practice was the only factor independently associated with opioid prescribing.

NSAID prescribing varied widely, likely reflecting conflicting evidence on the risk of anastomotic leaks [8, 9, 10]. Despite this concern, current guidelines recommend their use [6, 7] as the risk appears limited to specific agents (e.g., diclofenac) [10, 40]. Surgeons in Latin America/Caribbean, Southern and Western Europe, frequently prescribe dipyrone/metamizole for postoperative analgesia. While this drug is banned in the U.S., Canada, and the U.K. due to concerns about agranulocytosis [32], recent literature challenges the stigma around its use [32, 41]. Finally, non‐pharmacological interventions (e.g., ice‐packs, relaxation) were rarely prescribed by surgeons despite evidence supporting their potential benefits [42, 43], underscoring the need for greater awareness and integration of these strategies in perioperative care.

Our study has limitations inherent to survey‐based research. Snowball sampling may have introduced response bias, leading to clustered responses within regions, institutions, and networks of surgeons with greater interest in postoperative analgesia. This approach may also have led to uneven geographic dissemination, with greater representation from North American surgeons. Similarly, academic practitioners may be overrepresented and their prescribing practices may differ from those in non‐academic settings. The survey captured surgeons' self‐reported practices, which may not reflect real‐world prescribing behaviors due to reporting and recall bias. In some countries, surgeons do not determine the amount of opioids dispensed, limiting the analysis of prescribing amounts. The survey was open‐access, and preventive measures (i.e., captcha questions) may not have fully prevented invalid entries. As a cross‐sectional study, it reflects practices at a single time point and does not account for evolving guidelines or changes in prescribing over time. Differences in national opioid regulations, formularies, and drug availability may have influenced responses. Assessing the impact of specific non‐opioid analgesic approaches on opioid prescribing behaviors was beyond the scope of this study but remains an important area for future research. Finally, because the total number of surgeons reached through snowball sampling is unknown, a precise response rate (surveys completed/recipients) could not be calculated. Although participation rate (survey initiations/visitors = 84%) and completion rate (surveys completed/initiated = 80%), calculated according to CHERRIES guidelines [16], support strong engagement among respondents, these metrics do not reflect representativeness of the target population and should be interpreted accordingly [44].

As W. Edwards Deming, a pioneer in quality management, famously stated, “uncontrolled variation is the enemy of quality [45].” We hypothesize that the marked global variations in opioid prescribing are largely influenced by differences in care culture rather than patient need alone. The finding that 43% of participants manage post‐discharge pain without opioids highlights clinical equipoise and challenges the assumption that opioids are necessary for all patients undergoing colorectal resection. Clinically, our data provide international benchmarking that may prompt surgeons and institutions to re‐evaluate local prescribing practices. At a policy level, the observed variations underscore a lack of consensus and the need for further evidence to inform prescribing guidelines. From a research perspective, our results support the prioritization of robust comparative‐effectiveness trials evaluating opioid versus opioid‐free discharge analgesia after colorectal resections [11].

## 5 Conclusions

Findings from the ACORE survey revealed considerable global variations in opioid prescribing after elective colorectal resection. Notably, 43% of participants reported managing post‐discharge pain without opioids, underscoring clinical equipoise and challenging the assumption that opioids are universally necessary for all patients.

## Author Contributions

**Ghadeer Olleik:** conceptualization, methodology, investigation, data curation, formal analysis, writing – original draft, writing – review and editing. **Hiba Elhaj:** investigation, resources, writing – review and editing, data curation. **Samin Shirzadi:** investigation, data curation, writing – review and editing, resources. **Francesca Fermi:** investigation, data curation, writing – review and editing, formal analysis. **Maxime Lapointe‐Gagner:** investigation, data curation, writing – review and editing. **Sender Liberman:** investigation, data curation, writing – review and editing. **Mohsen Alhashemi:** investigation, data curation, writing – review and editing. **Tahereh Najafi Ghezeljeh:** investigation, data curation, writing – review and editing, resources. **Fatemeh Rajabiyazdi:** investigation, data curation, writing – review and editing. **Nawar Touma:** investigation, data curation, writing – review and editing. **Pepa Kaneva:** investigation, data curation, writing – review and editing. **Agnihotram V. Ramanakumar:** methodology, formal analysis, writing – review and editing, resources. **Badma Bashankaev:** investigation, data curation, writing – review and editing. **Alexandra Sidorova:** investigation, data curation, writing – review and editing. **Stephen J. Chapman:** investigation, data curation, writing – review and editing. **Chuan‐Gang Fu:** investigation, data curation, writing – review and editing. **Lucia Oliveira:** investigation, data curation, writing – review and editing. **Sofia Valanci:** investigation, data curation, writing – review and editing. **Audrius Dulskas:** investigation, writing – review and editing, data curation. **Steven Wexner:** conceptualization, investigation, writing – review and editing, supervision. **Lawrence Lee:** conceptualization, investigation, writing – review and editing, supervision. **Liane S. Feldman:** conceptualization, investigation, writing – review and editing, supervision. **Marylise Boutros:** conceptualization, investigation, writing – review and editing, supervision. **Julio F. Fiore Jr.:** conceptualization, methodology, investigation, formal analysis, supervision, writing – original draft, writing – review and editing.

## Funding

Ms. Olleik was supported by a studentship offered by Fonds de recherche du Québec‐Santé (FRQ‐S). Dr. Fiore received salary support from the Fonds de Recherche du Québec‐Santé (FRQ‐S). Funders had no role in the collection, analysis, interpretation of data, writing of the report, or decision to submit the article for publication.

## Ethics Statement

This study was approved by the McGill University ethics board (REB# A04‐E29‐19B).

## Consent

Informed consent was obtained from all individual participants included in the study.

## Conflicts of Interest

Ghadeer Olleik, Hiba Elhaj, Samin Shirzadi, Francesca Fermi, Maxime Lapointe‐Gagner, Mohsen Alhashemi, Tahereh Najafi Ghezeljeh, Fatemeh Rajabiyazdi, Nawar Touma, Pepa Kaneva, Agnihotram V. Ramanakumar, Alexandra Sidorova, Stephen J. Chapman, Chuan‐Gang Fu, Lucia Oliveira, Sofia Valanci, and Audrius Dulskas have no relevant disclosures or conflicts of interest. Sender Liberman has received research grants from Takeda and speaker fees from Abbott Nutrition. Steven Wexner is a consultant for ActivSurgical, Arthrex, Baxter, Becton Dickinson and Company, Intuitive Surgical, OstomyCure, Takeda, and Virtual Ports; receives royalties from Intuitive Surgical, Karl Storz Endoscopy America, and Unique Surgical Innovations; holds stock or stock options in GI View, OstomyCure, and Virtual Ports; and serves as chair of the data safety monitoring board for Polypid. Lawrence Lee is the recipient of an investigator‐initiated research grant from Johnson & Johnson and receives speaker fees from Stryker and Johnson & Johnson. Liane S. Feldman receives speaker fees and an investigator‐initiated research grant from Theator. Marylise Boutros receives a speaker honorarium from Johnson & Johnson. Julio F. Fiore Jr. is the recipient of an investigator‐initiated research grant from Merck.

## Compliance With Sex‐Inclusive SJEG Guidelines

Sex and gender were not relevant to this study, which examined surgeons' prescribing practices rather than patient‐level characteristics or outcomes. No sex‐ or gender‐based data were collected or analyzed.

## Supporting information


Supporting Information S1

## Data Availability

The data that support the findings of this study are available from the corresponding author upon reasonable request.

## References

[1] M. D. Neuman , B. T. Bateman , and H. Wunsch , “Inappropriate Opioid Prescription After Surgery,” Lancet 393, no. 10180 (2019): 1547–1557, 10.1016/s0140-6736(19)30428-3. 30983590 PMC6556783

[2] K. Humphreys , C. L. Shover , C. M. Andrews , et al., “Responding to the Opioid Crisis in North America and Beyond: Recommendations of the Stanford‐Lancet Commission,” Lancet 399, no. 10324 (2022): 555–604, 10.1016/s0140-6736(21)02252-2. 35122753 PMC9261968

[3] C. M. Brummett , J. F. Waljee , J. Goesling , et al., “New Persistent Opioid Use After Minor and Major Surgical Procedures in US Adults,” JAMA Surgery 152, no. 6 (2017): e170504, 10.1001/jamasurg.2017.0504. 28403427 PMC7050825

[4] A. C. Fields , P. M. Cavallaro , D. J. Correll , et al., “Predictors of Prolonged Opioid Use Following Colectomy,” Diseases of the Colon & Rectum 62, no. 9 (2019): 1117–1123, 10.1097/dcr.0000000000001429. 31318765

[5] S. J. Chapman , A. Pericleous , C. Downey , and D. G. Jayne , “Postoperative Ileus Following Major Colorectal Surgery,” British Journal of Surgery 105, no. 7 (2018): 797–810, 10.1002/bjs.10781. 29469195

[6] J. L. Irani , T. L. Hedrick , T. E. Miller , et al., “Clinical Practice Guidelines for Enhanced Recovery After Colon and Rectal Surgery From the American Society of Colon and Rectal Surgeons and the Society of American Gastrointestinal and Endoscopic Surgeons,” Surgical Endoscopy 37, no. 1 (2023): 5–30, 10.1007/s00464-022-09758-x. 36515747 PMC9839829

[7] U. O. Gustafsson , M. J. Scott , M. Hubner , et al., “Guidelines for Perioperative Care in Elective Colorectal Surgery: Enhanced Recovery After Surgery (ERAS(®)) Society Recommendations: 2018,” World Journal of Surgery 43, no. 3 (2019): 659–695, 10.1007/s00268-018-4844-y. 30426190

[8] M. Klein , I. Gögenur , and J. Rosenberg , “Postoperative Use of Non‐Steroidal Anti‐Inflammatory Drugs in Patients With Anastomotic Leakage Requiring Reoperation After Colorectal Resection: Cohort Study Based on Prospective Data,” BMJ 345, no. 3 (2012): e6166, 10.1136/bmj.e6166. 23015299 PMC3458793

[9] T. W. Hakkarainen , S. R. Steele , A. Bastaworous , et al., “Nonsteroidal Anti‐Inflammatory Drugs and the Risk for Anastomotic Failure: A Report From Washington State’s Surgical Care and Outcomes Assessment Program (SCOAP),” JAMA Surgery 150, no. 3 (2015): 223–228, 10.1001/jamasurg.2014.2239. 25607250 PMC4524521

[10] EuroSurg Collaborative , “Safety and Efficacy of Non‐Steroidal Anti‐Inflammatory Drugs to Reduce Ileus After Colorectal Surgery,” British Journal of Surgery 107, no. 2 (2020): e161–e169, PMID: 31595986, 10.1002/bjs.11326. 31595986

[11] J. F. Fiore Jr. , C. El‐Kefraoui , M. A. Chay , et al., “Opioid Versus Opioid‐Free Analgesia After Surgical Discharge: A Systematic Review and Meta‐Analysis of Randomised Trials,” Lancet 399, no. 10343 (2022): 2280–2293, 10.1016/s0140-6736(22)00582-7. 35717988

[12] K. Kelley , B. Clark , V. Brown , and J. Sitzia , “Good Practice in the Conduct and Reporting of Survey Research,” International Journal for Quality in Health Care 15, no. 3 (2003): 261–266, 10.1093/intqhc/mzg031. 12803354

[13] K. E. Burns , M. Duffett , M. E. Kho , et al., “A Guide for the Design and Conduct of Self‐Administered Surveys of Clinicians,” Canadian Medical Association Journal 179, no. 3 (2008): 245–252, 10.1503/cmaj.080372. 18663204 PMC2474876

[14] K. Brasel , A. Haider , and J. Haukoos , “Practical Guide to Survey Research,” JAMA Surgery 155, no. 4 (2020): 351–352, 10.1001/jamasurg.2019.4401. 31995149

[15] A. Sharma , N. T. Minh Duc , T. T. Luu Lam , et al., “A Consensus‐Based Checklist for Reporting of Survey Studies (CROSS),” Journal of General Internal Medicine 36, no. 10 (2021): 3179–3187, 10.1007/s11606-021-06737-1. 33886027 PMC8481359

[16] G. Eysenbach , “Improving the Quality of Web Surveys: The Checklist for Reporting Results of Internet E‐Surveys (CHERRIES),” Journal of Medical Internet Research 6, no. 3 (2004): e34, 10.2196/jmir.6.3.e34. 15471760 PMC1550605

[17] J. F. Fiore Jr. , G. Olleik , C. El‐Kefraoui , et al., “Preventing Opioid Prescription After Major Surgery: A Scoping Review of Opioid‐Free Analgesia,” British Journal of Anaesthesia 123, no. 5 (2019): 627–636, 10.1016/j.bja.2019.08.014. 31563269

[18] Ghewgill , “Language Graphs and Areas,” Accessed, June 22, 2022, https://hewgill.com/language‐graphs/language‐areas.html.

[19] P. Edwards , I. Roberts , P. Sandercock , and C. Frost , “Follow‐up by Mail in Clinical Trials: Does Questionnaire Length Matter?,” Controlled Clinical Trials 25, no. 1 (2004): 31–52, 10.1016/j.cct.2003.08.013. 14980747

[20] P. J. Edwards , I. Roberts , M. J. Clarke , et al., “Methods to Increase Response to Postal and Electronic Questionnaires,” Cochrane Database of Systematic Reviews 2009, no. 11 (2009): Mr000008, 10.1002/14651858.MR000008.pub5. 38032037 PMC10687884

[21] P. Sedgwick , “Snowball Sampling,” BMJ 347, no. dec20 2 (2013): f7511, 10.1136/bmj.f7511.

[22] P. H. Rossi , J. D. Wright , and A. B. Anderson , eds., Handbook of Survey Research. 2nd ed. (Academic Press, 2013).

[23] H. M. A. Kaafarani , K. Han , M. El Moheb , et al., “Opioids After Surgery in the United States Versus the Rest of the World: The International Patterns of Opioid Prescribing (iPOP) Multicenter Study,” Annals of Surgery 272, no. 6 (2020): 879–886, 10.1097/sla.0000000000004225. 32657939

[24] D. S. Keller and F. D. McDermott , “Social Media and the Evolution of Colorectal Disease,” Colorectal Disease 22, no. 2 (2020): 127–128, 10.1111/codi.14974. 32017370

[25] V. K. Podichetty , J. Booher , M. Whitfield , and R. S. Biscup , “Assessment of Internet Use and Effects Among Healthcare Professionals: A Cross Sectional Survey,” Postgraduate Medical Journal 82, no. 966 (2006): 274–279, 10.1136/pgmj.2005.040675. 16597816 PMC2579634

[26] United Nations Statistics Division , “United Nations Classification for Geographic Regions,” Accessed June 22, 2022, https://unstats.un.org/unsd/methodology/m49/.

[27] Utah Department of Health , “Opioid Oral Morphine Equivalent (MME) Conversion Factors,” (2021), https://medicaid.utah.gov/Documents/files/Opioid‐Morphine‐EQ‐Conversion‐Factors.pdf.

[28] A. Genell , S. Nemes , G. Steineck , and P. W. Dickman , “Model Selection in Medical Research: A Simulation Study Comparing Bayesian Model Averaging and Stepwise Regression,” BMC Medical Research Methodology 10, no. 1 (2010): 108, 10.1186/1471-2288-10-108. 21134252 PMC3017523

[29] J. A. Hoeting , D. Madigan , A. E. Raftery , and C. T. Volinsky , “Bayesian Model Averaging: A Tutorial,” Statistical Science 14, no. 4(1999): 382–401, http://www.jstor.org/stable/2676803.

[30] D. Wang , W. Zhang , and A. Bakhai , “Comparison of Bayesian Model Averaging and Stepwise Methods for Model Selection in Logistic Regression,” Statistics in Medicine 23, no. 22 (2004): 3451–3467, 10.1002/sim.1930. 15505893

[31] R. E. Kass , and A. E. Raftery , “Bayes Factors,” Journal of the American Statistical Association 90, no. 430 (1995): 773–795, 10.2307/2291091.

[32] M. N. Miljković , N. K. Rančić , R. M. Simić , D. M. Stamenković , and V. M. Dragojević‐Simić , “Metamizole: Current Status of the Safety and Efficacy,” Hospital Pharmacology 5, no. 3 (2018): 694–704, 10.5937/hpimj1803694M.

[33] K. S. Ladha , M. D. Neuman , G. Broms , et al., “Opioid Prescribing After Surgery in the United States, Canada, and Sweden,” JAMA Network Open 2, no. 9 (2019): e1910734, 10.1001/jamanetworkopen.2019.10734. 31483475 PMC6727684

[34] R. J. Li , M. Loyo Li , E. Leon , et al., “Comparison of Opioid Utilization Patterns After Major Head and Neck Procedures Between Hong Kong and the United States,” JAMA Otolaryngology—Head & Neck Surgery 144, no. 11 (2018): 1060–1065, 10.1001/jamaoto.2018.1787. 30193293 PMC6248185

[35] A. M. Weyh , R. Pucci , E. Busby , et al., “Contrasting Opioid Use for Pain Management in Microvascular Head and Neck Reconstruction: An International Study,” International Journal of Oral and Maxillofacial Surgery 51, no. 11 (2022): 1412–1419, 10.1016/j.ijom.2022.04.016. 35599083

[36] H. J. Curtis , R. Croker , A. J. Walker , G. C. Richards , J. Quinlan , and B. Goldacre , “Opioid Prescribing Trends and Geographical Variation in England, 1998–2018: A Retrospective Database Study,” Lancet Psychiatry 6, no. 2 (2019): 140–150, 10.1016/s2215-0366(18)30471-1. 30580987

[37] B. Blanch , S. A. Pearson , and P. S. Haber , “An Overview of the Patterns of Prescription Opioid Use, Costs and Related Harms in Australia,” British Journal of Clinical Pharmacology 78, no. 5 (2014): 1159–1166, 10.1111/bcp.12446. 24962372 PMC4243891

[38] P. Böckerman , M. Haapanen , C. Hakulinen , H. Karhunen , and T. Maczulskij , “Determinants of Prescription Opioid Use: Population‐Based Evidence From Finland,” Addiction 116, no. 1 (2021): 170–175, 10.1111/add.15071. 32267581

[39] S. Peacock and S. Patel , “Cultural Influences on Pain,” Reviews in pain 1, no. 2 (2008): 6–9, 10.1177/204946370800100203. 26525084 PMC4589930

[40] A. Modasi , D. Pace , M. Godwin , C. Smith , and B. Curtis , “NSAID Administration Post Colorectal Surgery Increases Anastomotic Leak Rate: Systematic Review/Meta‐Analysis,” Surgical Endoscopy 33, no. 3 (2019): 879–885, 10.1007/s00464-018-6355-1. 29998389

[41] J. Konijnenbelt‐Peters , C. van der Heijden , C. Ekhart , J. Bos , J. Bruhn , and C. Kramers , “Metamizole (Dipyrone) as an Alternative Agent in Postoperative Analgesia in Patients With Contraindications for Nonsteroidal Anti‐Inflammatory Drugs,” Pain Practice 17, no. 3 (2017): 402–408, 10.1111/papr.12467. 27346584

[42] H. Muaddi , E. Lillie , S. Silva , et al., “The Effect of Cryotherapy Application on Postoperative Pain: A Systematic Review and Meta‐analysis,” Annals of Surgery 277, no. 2 (2023): e257–e265, 10.1097/sla.0000000000004987. 34856580

[43] A. Y. R. Kühlmann , A. de Rooij , L. F. Kroese , M. van Dijk , M. G. M. Hunink , and J. Jeekel , “Meta‐Analysis Evaluating Music Interventions for Anxiety and Pain in Surgery,” British Journal of Surgery 105, no. 7 (2018): 773–783, 10.1002/bjs.10853. 29665028 PMC6175460

[44] P. D. Welsby , “Surveys in Decline: The Death of the Denominator,” Postgraduate Medical Journal 98, no. 1166 (2022): e44, 10.1136/postgradmedj-2020-139406. 33790038

[45] D. W. Edwards , Basic Statistical Tools for Improving Quality, C. W. Kang and P. H. Kvam , eds. (Wiley, 2012), 19.

