# Supplementary material for: Post‐Discharge Opioid Prescribing After Elective Colorectal Resection: An International Survey
Source: World J Surg. 2026 Jan 29;50(3):558–68. doi: 10.1002/wjs.70245 (PMC13006777; doi:10.1002/wjs.70245)
Supplement: Supplementary file 1 — Supporting Information S1 [file WJS-50-558-s001.docx]

**Supplementary Material**

**Post-discharge opioid prescribing after elective colorectal resection: An international survey**

Ghadeer Olleik MSc; Hiba Elhaj MSc; Samin Shirzadi MD MPH; Francesca Fermi MD; Maxime Lapointe-Gagner MSc; Sender Liberman MD; Mohsen Alhashemi MD MSc ; Tahereh Najafi Ghezeljeh PhD; Fatemeh Rajabiyazdi PhD; Nawar Touma MSc^1^; Pepa Kaneva MSc; Agnihotram V. Ramanakumar, PhD; Badma Bashankaev MD; Alexandra Sidorova MD; Stephen J Chapman MD PhD; Chuan-Gang Fu MD PhD; Lucia Oliveira MD PhD; Sofia Valanci MD PhD; Audrius Dulskas MD; Steven Wexner MD PhD; Lawrence Lee MD PhD; Liane S Feldman MD; Marylise Boutros MD; Julio F Fiore Jr. PhD*

**Department of Surgery, McGill University, Montreal, QC, Canada. Email: julio.fiorejunior@mcgill.ca*

Table of Contents

[Table S1. Checklist for Reporting of Survey Studies (CROSS) 2](#_Toc200406244)

[Table S2. Checklist for Reporting Results of Internet E-Surveys (CHERRIES) 4](#_Toc200406245)

[Table S3. Analgesia prescribing at hospital discharge after elective colorectal resection: An international survey (Translated to French, Arabic, Spanish, Portuguese, Russian, Mandarin Chinese) 7](#_Toc200406246)

[Figure S1. X platform content used for survey distribution 15](#_Toc200406247)

[Figure S2. Participant flowchart 16](#_Toc200406248)

[Table S4. Post-discharge opioid prescribing practices across different continental region, subregion, and countries (n=817) 17](#_Toc200406249)

[Table S5. In-hospital care characteristics in different world subregions 21](#_Toc200406253)

[Table S6. Opioid medications prescribed at discharge in different world subregions 28](#_Toc200406260)

[Table S7. Total Morphine Milligram Equivalents (MME) prescribed in different subregions (sensitivity analysis) 32](#_Toc200406264)

[Table S8. Non-opioid medications prescribed at discharge in different world subregions 33](#_Toc200406265)

[Table S9. NSAIDs medications prescribed at discharge in different world subregions 35](#_Toc200406266)

[Table S10. Bayesian model averaging (BMA) analysis of potential predictors opioid prescribing at discharge after minimally invasive surgery (Multiple BMA analysis) (n=760) 37](#_Toc200406268)

[Table S11. Bayesian model averaging (BMA) analysis of potential predictors of opioid prescribing at discharge after open surgery (Multiple BMA analysis) (n=640) 38](#_Toc200406269)

# Table S1. Checklist for Reporting of Survey Studies (CROSS)

| **Section/topic** | **Item** | **Item description** | **Reported on page #** |
| --- | --- | --- | --- |
| **Title and abstract** | | |  |
| Title and abstract | 1a | State the word “survey” along with a commonly used term in title or abstract to introduce the study’s design. | 1,3 |
|  | 1b | Provide an informative summary in the abstract, covering background, objectives, methods, findings/results, interpretation/discussion, and conclusions. | 3 |
| **Introduction** | | |  |
| Background | 2 | Provide a background about the rationale of study, what has been previously done, and why this survey is needed. | 5 |
| Purpose/aim | 3 | Identify specific purposes, aims, goals, or objectives of the study. | 5 |
| **Methods** | | |  |
| Study design | 4 | Specify the study design in the methods section with a commonly used term (e.g., cross-sectional or longitudinal). | 5,6 |
|  | 5a | Describe the questionnaire (e.g., number of sections, number of questions, number and names of instruments used). | 6,7 |
| Data collection methods | 5b | Describe all questionnaire instruments that were used in the survey to measure particular concepts. Report target population, reported validity and reliability information, scoring/classification procedure, and reference links (if any). | 6,7,8 |
|  | 5c | Provide information on pretesting of the questionnaire, if performed (in the article or in an online supplement). Report the method of pretesting, number of times questionnaire was pre-tested, number and demographics of participants used for pretesting, and the level of similarity of demographics between pre-testing participants and sample population. | 6,7 |
|  | 5d | Questionnaire if possible, should be fully provided (in the article, or as appendices or as an online supplement). | Table S3 |
| Sample characteristics | 6a | Describe the study population (i.e., background, locations, eligibility criteria for participant inclusion in survey, exclusion criteria). | 7 |
|  | 6b | Describe the sampling techniques used (e.g., single stage or multistage sampling, simple random sampling, stratified sampling, cluster sampling, convenience sampling). Specify the locations of sample participants whenever clustered sampling was applied. | 7,8 |
|  | 6c | Provide information on sample size, along with details of sample size calculation. | N/A |
|  | 6d | Describe how representative the sample is of the study population (or target population if possible), particularly for population-based surveys. | 6,7 |
| Survey  administration | 7a | Provide information on modes of questionnaire administration, including the type and number of contacts, the location where the survey was conducted (e.g., outpatient room or by use of online tools, such as SurveyMonkey). | 6,7,8 |
|  | 7b | Provide information of survey’s time frame, such as periods of recruitment, exposure, and follow-up days. | 9 |
|  | 7c | Provide information on the entry process:  –>For non-web-based surveys, provide approaches to minimize human error in data entry.  –>For web-based surveys, provide approaches to prevent “multiple participation” of participants. | 6,7 |
| Study preparation | 8 | Describe any preparation process before conducting the survey (e.g., interviewers’ training process, advertising the survey). | 6,7,8 |
| Ethical considerations | 9a | Provide information on ethical approval for the survey if obtained, including informed consent, institutional review board [IRB] approval, Helsinki declaration, and good clinical practice [GCP] declaration (as appropriate). | 5 |
|  | 9b | Provide information about survey anonymity and confidentiality and describe what mechanisms were used to protect unauthorized access. | 7, Table S3 |
| Statistical  analysis | 10a | Describe statistical methods and analytical approach. Report the statistical software that was used for data analysis. | 8,9 |
|  | 10b | Report any modification of variables used in the analysis, along with reference (if available). | N/A |
|  | 10c | Report details about how missing data was handled. Include rate of missing items, missing data mechanism (i.e., missing completely at random [MCAR], missing at random [MAR] or missing not at random [MNAR]) and methods used to deal with missing data (e.g., multiple imputation). | 8,9 |
|  | 10d | State how non-response error was addressed. | 6,7 |
|  | 10e | For longitudinal surveys, state how loss to follow-up was addressed. | N/A |
|  | 10f | Indicate whether any methods such as weighting of items or propensity scores have been used to adjust for non-representativeness of the sample. | N/A |
|  | 10g | Describe any sensitivity analysis conducted. | 9 |
| **Results** | | |  |
| Respondent characteristics | 11a | Report numbers of individuals at each stage of the study. Consider using a flow diagram, if possible. | 9,10 Figure S2 |
|  | 11b | Provide reasons for non-participation at each stage, if possible. | 9,10 Figure S2 |
|  | 11c | Report response rate, present the definition of response rate or the formula used to calculate response rate. | 8,9,10 |
|  | 11d | Provide information to define how unique visitors are determined. Report number of unique visitors along with relevant proportions (e.g., view proportion, participation proportion, completion proportion). | 8,9,10 Table S2 |
| Descriptive  results | 12 | Provide characteristics of study participants, as well as information on potential confounders and assessed outcomes. | N/A |
| Main findings | 13a | Give unadjusted estimates and, if applicable, confounder-adjusted estimates along with 95% confidence intervals and p-values. | N/A |
|  | 13b | For multivariable analysis, provide information on the model building process, model fit statistics, and model assumptions (as appropriate). | 9,13 |
|  | 13c | Provide details about any sensitivity analysis performed. If there are considerable amount of missing data, report sensitivity analyses comparing the results of complete cases with that of the imputed dataset (if possible). | 9,12 |
| **Discussion** | | |  |
| Limitations | 14 | Discuss the limitations of the study, considering sources of potential biases and imprecisions, such as non-representativeness of sample, study design, important uncontrolled confounders. | 15, 16 |
| Interpretations | 15 | Give a cautious overall interpretation of results, based on potential biases and imprecisions and suggest areas for future research. | 13, 14, 15, 16 |
| Generalizability | 16 | Discuss the external validity of the results. | 15, 16 |
| **Other sections** | | |  |
| Role of funding source | 17 | State whether any funding organization has had any roles in the survey’s design, implementation, and analysis. | 2 |
| Conflict of interest | 18 | Declare any potential conflict of interest. | 2 |
| Acknowledgements | 19 | Provide names of organizations/persons that are acknowledged along with their contribution to the research. | 17, 18 |

# Table S2. Checklist for Reporting Results of Internet E-Surveys (CHERRIES)

| **Item Category** | **Checklist Item** | **Explanation** | **Reported on page** |
| --- | --- | --- | --- |
| **Design** | Describe survey design | - Describe target population, sample frame. Is the sample a convenience sample? (In “open” surveys this is most likely.) | 6,7 |
| - **IRB (Institutional Review Board) approval and informed consent process** | | |  |
|  | IRB approval | - Mention whether the study has been approved by an IRB. | 5 |
|  | Informed consent | - Describe the informed consent process. Where were the participants told the length of time of the survey, which data were stored and where and for how long, who the investigator was, and the purpose of the study? | 5,  Table S3 |
|  | Data protection | - If any personal information was collected or stored, describe what mechanisms were used to protect unauthorized access. | 6,7 |
| - **Development and pre-testing** | | |  |
|  | Development and testing | - State how the survey was developed, including whether the usability and technical functionality of the electronic questionnaire had been tested before fielding the questionnaire. | 6,7 |
| - **Recruitment process and description of the sample having access to the questionnaire** | | |  |
|  | Open survey versus closed survey | - An “open survey” is a survey open for each visitor of a site, while a closed survey is only open to a sample which the investigator knows (password-protected survey). | 6,7 |
|  | Contact mode | - Indicate whether or not the initial contact with the potential participants was made on the Internet. (Investigators may also send out questionnaires by mail and allow for Web-based data entry.) | 7,8 |
|  | Advertising the survey | - How/where was the survey announced or advertised? Some examples are offline media (newspapers), or online (mailing lists – If yes, which ones?) or banner ads (Where were these banner ads posted and what did they look like?). It is important to know the wording of the announcement as it will heavily influence who chooses to participate. Ideally the survey announcement should be published as an appendix. | 7,8,  Figure S1 |
| - **Survey administration** | | |  |
|  | Web/E-mail | - State the type of e-survey (eg, one posted on a Web site, or one sent out through e-mail). If it is an e-mail survey, were the responses entered manually into a database, or was there an automatic method for capturing responses? | 7,8  Figure S1 |
|  | Context | - Describe the Web site (for mailing list/newsgroup) in which the survey was posted. What is the Web site about, who is visiting it, what are visitors normally looking for? Discuss to what degree the content of the Web site could pre-select the sample or influence the results. For example, a survey about vaccination on a anti-immunization Web site will have different results from a Web survey conducted on a government Web site | 6,7,8 |
|  | Mandatory/voluntary | - Was it a mandatory survey to be filled in by every visitor who wanted to enter the Web site, or was it a voluntary survey? | 6,7 |
|  | Incentives | - Were any incentives offered (eg, monetary, prizes, or non-monetary incentives such as an offer to provide the survey results)? | N/A |
|  | Time/Date | - In what timeframe were the data collected? | 9 |
|  | Randomization of items or questionnaires | - To prevent biases items can be randomized or alternated. | N/A |
|  | Adaptive questioning | - Use adaptive questioning (certain items, or only conditionally displayed based on responses to other items) to reduce number and complexity of the questions. | 6 |
|  | Number of Items | - What was the number of questionnaire items per page? The number of items is an important factor for the completion rate. | 6,7,  Table S3 |
|  | Number of screens (pages) | - Over how many pages was the questionnaire distributed? The number of items is an important factor for the completion rate. | 6,  Table S3 |
|  | Completeness check | - It is technically possible to do consistency or completeness checks before the questionnaire is submitted. Was this done, and if “yes”, how (usually JAVAScript)? An alternative is to check for completeness after the questionnaire has been submitted (and highlight mandatory items). If this has been done, it should be reported. All items should provide a non-response option such as “not applicable” or “rather not say”, and selection of one response option should be enforced. | 6,7 |
|  | Review step | - State whether respondents were able to review and change their answers (eg, through a Back button or a Review step which displays a summary of the responses and asks the respondents if they are correct). | 7 |
| - **Response rates** | | |  |
|  | Unique site visitor | - If you provide view rates or participation rates, you need to define how you determined a unique visitor. There are different techniques available, based on IP addresses or cookies or both. | N/A |
|  | View rate (Ratio of unique survey visitors/unique site visitors) | - Requires counting unique visitors to the first page of the survey, divided by the number of unique site visitors (not page views!). It is not unusual to have view rates of less than 0.1 % if the survey is voluntary. | N/A |
|  | Participation rate (Ratio of unique visitors who agreed to participate/unique first survey page visitors) | - Count the unique number of people who filled in the first survey page (or agreed to participate, for example by checking a checkbox), divided by visitors who visit the first page of the survey (or the informed consents page, if present). This can also be called “recruitment” rate. | 8,9,10 |
|  | Completion rate (Ratio of users who finished the survey/users who agreed to participate) | - The number of people submitting the last questionnaire page, divided by the number of people who agreed to participate (or submitted the first survey page). This is only relevant if there is a separate “informed consent” page or if the survey goes over several pages. This is a measure for attrition. Note that “completion” can involve leaving questionnaire items blank. This is not a measure for how completely questionnaires were filled in. (If you need a measure for this, use the word “completeness rate”.) | 8,9,10 |
| - **Preventing multiple entries from the same individual** | | |  |
|  | Cookies used | - Indicate whether cookies were used to assign a unique user identifier to each client computer. If so, mention the page on which the cookie was set and read, and how long the cookie was valid. Were duplicate entries avoided by preventing users access to the survey twice; or were duplicate database entries having the same user ID eliminated before analysis? In the latter case, which entries were kept for analysis (eg, the first entry or the most recent)? | 7 |
|  | IP check | - Indicate whether the IP address of the client computer was used to identify potential duplicate entries from the same user. If so, mention the period of time for which no two entries from the same IP address were allowed (eg, 24 hours). Were duplicate entries avoided by preventing users with the same IP address access to the survey twice; or were duplicate database entries having the same IP address within a given period of time eliminated before analysis? If the latter, which entries were kept for analysis (eg, the first entry or the most recent)? | N/A |
|  | Log file analysis | - Indicate whether other techniques to analyze the log file for identification of multiple entries were used. If so, please describe. | N/A |
|  | Registration | - In “closed” (non-open) surveys, users need to login first and it is easier to prevent duplicate entries from the same user. Describe how this was done. For example, was the survey never displayed a second time once the user had filled it in, or was the username stored together with the survey results and later eliminated? If the latter, which entries were kept for analysis (eg, the first entry or the most recent)? | N/A |
| - **Analysis** | | |  |
|  | Handling of incomplete questionnaires | - Were only completed questionnaires analyzed? Were questionnaires which terminated early (where, for example, users did not go through all questionnaire pages) also analyzed? | 8,9 |
|  | Questionnaires submitted with an atypical timestamp | - Some investigators may measure the time people needed to fill in a questionnaire and exclude questionnaires that were submitted too soon. Specify the timeframe that was used as a cut-off point, and describe how this point was determined. | N/A |
|  | Statistical correction | - Indicate whether any methods such as weighting of items or propensity scores have been used to adjust for the non-representative sample; if so, please describe the methods. | N/A |

# Table S3. Analgesia prescribing at hospital discharge after elective colorectal resection: An international survey (Translated to French, Arabic, Spanish, Portuguese, Russian, Mandarin Chinese)

We appreciate your participation in this survey study, which aims to **characterize international patterns of analgesia prescription** **following elective colorectal resection**. In light of the opioid crisis currently affecting colorectal surgery patients in many countries, findings from this study will help direct care and future research on postoperative pain management of this patient population.

The survey should take approximately **5 minutes** to complete. Your responses are anonymous and survey completion will be considered as your consent to participate. For any questions or comments about this study, please contact: pcorlab.surgery@mcgill.ca

*While responding to the survey, please keep in mind that:*

- *For the purpose of this study, elective colorectal resection is defined as the removal of parts of the colon and/or rectum undertaken via an abdominal approach (open, Minimally invasive, robotic or hybrid) on a planned basis.*
- *To reduce heterogeneity, our survey does not account for anorectal procedures (e.g., hemorrhoidectomy, fistulotomy), procedures involving a perineal incision (e.g., abdominal perineal resection), or transanal surgeries (e.g., local excisions of rectal polyps, transanal endoscopic surgery).*

Please click ‘Next’ to continue

| 1. **Please select the statement that best reflects your surgical activities:**   **Elective colorectal resection: the removal of parts of the colon and/or rectum via an abdominal approach undertaken on a planned basis.* | - I perform elective colorectal resections using both open and minimally invasive approaches (i.e., Minimally invasive, robotic). - I only perform elective colorectal resections using an open approach. - I only perform elective colorectal resections using minimally invasive approaches (unless converted to open). - I **DO NOT** perform elective colorectal resections in my practice.   (if last choice is chosen the following message will show: Thank you for your interest in this survey; however, we are looking for information from **surgeons and/or trainees** who**perform colorectal resections** in their practice.  Please click 'Next' to finish this survey. If you have any suggestions or important information that you would like to share, please feel free to include them in the space below.) |
| --- | --- |
| 1. **Please select what best reflects your current position:**   *Practicing surgeon: A physician who practices surgery independently, unsupervised. | - Practicing colorectal surgeon/proctologist * - Practicing gastrointestinal surgeon * - Practicing general surgeon * - Senior resident/senior registrar/fellow (>4 years of surgical training) - Junior resident/junior registrar (≤3 years of surgical training) |
| 1. **Approximately how many years of independent practice do you have?**   *If respondent selected ‘colorectal surgeon/proctologist’, ‘gastrointestinal surgeon’ or ‘general surgeon’ in question 2 |  <3   3-5   6-10   11-15   16-20   21-25   26-30   31-35   36-40   >40 |
| 1. **In which country did you complete the majority of your surgical training** (i.e., residency, fellowship)?   *If respondent selected ‘colorectal surgeon/proctologist’, ‘gastrointestinal surgeon’ or ‘general surgeon’ in question 2 | A list will appear, and one answer can be chosen |
| 1. **In which country do you currently practice/train?** | A list will appear, and one answer can be chosen |
| 1. **What best characterizes your predominant practice/training location?**   *Please select ALL that apply. | - Academic hospital (university-affiliated) - Community hospital (not university-affiliated) - Private practice/hospital - Other, please specify: _________________ |
| 1. **Approximately how many elective colorectal resections do you perform annually?**   **Elective colorectal resection: the removal of parts of the colon and/or rectum via an abdominal approach undertaken on a planned basis.* | - 1-10 - 11-30 - 31-60 - 61-100 - >100 |
| 1. **Please provide any further comments in the box below:** |  |

**Analgesia during hospital stay**

The questions below focus on your analgesia practice **during hospital stay** considering a typical patient who did not develop intra- or post-operative complications:

| 1. **Are colorectal surgery patients in your practice treated using an enhanced recovery (ERAS, fast-track) pathway?**   ** Enhanced recovery (ERAS, fast-track) pathways: multimodal perioperative care programs aimed to improve postoperative recovery.* | - Yes - No |
| --- | --- |
| 1. **On average, patients undergoing open colorectal resection in your practice stay in the hospital for how many days?**   *Please provide the best possible estimate based on the majority of the patients in your practice.  *If respondent selected open or both (open and minimally invasive). | A drop-down menu will appear, and one answer can be chosen |
| 1. **Most patients undergoing open colorectal resection receive in-hospital analgesia via the following methods:**   *Please select ALL that apply.  *If respondent selected open surgery or both (open and minimally invasive). | - Peripheral nerve block [e.g., Transversus abdominis plane (TAP) block] - Wound infiltration - Epidural analgesia - Spinal analgesia - Intravenous (IV) analgesia [including IV patient-controlled analgesia (PCA)] - Oral analgesia - Rectal analgesia - Other, please specify: __________________ |
| 1. **Most patients undergoing open colorectal resection receive in-hospital analgesia using the following types/classes of drugs:**   *Please select ALL that apply.  *If respondent selected open surgery or both (open and minimally invasive). | - Opioids (e.g., Morphine, Fentanyl, Oxycodone) - Traditional local anesthetics (e.g., Lidocaine, Bupivacaine, Ropivacaine) - Sustained release local anesthetics (e.g., Liposomal Bupivacaine) - Acetaminophen/Paracetamol, intravenous - Acetaminophen/Paracetamol, oral - Non-steroidal anti-inflammatory drugs (e.g., Ibuprofen, Ketorolac, Celecoxib) - Dipyrone/Metamizole - Gabapentinoids - Dexmedetomidine - Nefopam - Clonidine - Ketamine - Other, please specify: __________________ |
| 1. **On average, patients undergoing minimally invasive colorectal resection in your practice stay in the hospital for how many days?**   *Please provide the best possible estimate based on the majority of the patients in your practice.  *****If respondent selected minimally invasive surgery or both (open and minimally invasive). | A drop-down menu will appear, and one answer can be chosen |
| 1. **The in-hospital analgesia used in your practice:**   *****If respondent selects both (open and minimally invasive). | - Is the **same** for patients undergoing open or minimally invasive surgery - Is different between patients undergoing open or minimally invasive surgery |
| 1. **Most patients undergoing minimally invasive colorectal resection receive in-hospital analgesia via the following methods:**   *Please select ALL that apply.  *If respondent selected minimally invasive surgery or both (open and minimally invasive) and in-hospital analgesia is different. | - Peripheral nerve block [e.g., Transversus abdominis plane (TAP) block] - Wound infiltration - Epidural analgesia - Spinal analgesia - Intravenous (IV) analgesia [including IV patient-controlled analgesia (PCA)] - Oral analgesia - Rectal analgesia - Other, please specify: __________________ |
| 1. **Most patients undergoing minimally invasive colorectal resection receive in-hospital analgesia using the following types/classes of drugs:**   *Please select ALL that apply.  **If respondent selected minimally invasive or both (open and minimally invasive) surgery and in-hospital analgesia is different. | - Opioids (e.g., Morphine, Fentanyl, Oxycodone) - Traditional local anesthetics (e.g., Lidocaine, Bupivacaine, Ropivacaine) - Sustained release local anesthetics (e.g., Liposomal Bupivacaine) - Acetaminophen/Paracetamol, intravenous - Acetaminophen/Paracetamol, oral - Non-steroidal anti-inflammatory drugs (e.g., Ibuprofen, Ketorolac, Celecoxib) - Dipyrone/Metamizole - Gabapentinoids - Dexmedetomidine - Nefopam - Clonidine - Ketamine - Other, please specify: __________________ |
| 1. **Which non-steroidal anti-inflammatory (NSAID) drug/drugs do you usually prescribe for in-hospital analgesia?**   *Please select ALL that apply.  **This information is relevant for a nested study focused on in-hospital use of non-steroidal anti-inflammatory drugs after colorectal resection.  *If respondent selected ‘non-steroidal anti-inflammatory drugs’ when describing in-hospital analgesia for open and/or minimally invasive surgery. | - Ibuprofen - Naproxen - Diclofenac - Celecoxib - Ketorolac - Ketoprofen - Etodolac - Indomethacin - Meloxicam - Piroxicam - Other, please specify: __________________ |
| 1. **Please provide any further comments in the box below:** |  |

**Analgesia prescribing at hospital discharge**

The questions below focus on your analgesia practice **at hospital discharge** considering a typical patient who did not develop intra- or post-operative complications:

| 1. **Which analgesic medications do you usually prescribe for open colorectal resection at hospital discharge?**   *Please select ALL that apply.  *If you prescribe medications that combine two different drugs in the same pill (e.g., acetaminophen + codeine, acetaminophen + oxycodone), please select all the drugs included in the combination.  *****This will appear based on respondents’ choices whether performing open or both (open and minimally invasive). | - Acetaminophen/Paracetamol - Ibuprofen - Naproxen - Diclofenac - Ketorolac - Celecoxib - Dipyrone/Metamizole - Gabapentinoids - Nefopam - Codeine - Hydrocodone - Nalbuphine - Hydromorphone - Morphine - Meperidine - Oxycodone (regular, immediate release) - Oxycodone (extended, slow release) - Tapentadol - Tramadol - Methadone - Transdermal opioids (e.g., fentanyl/morphine) - Other, please specify: __________________ - Analgesic medications are usually not prescribed at discharge (for example, due to long hospital stay) |
| --- | --- |
| 1. **How are patients having open colorectal resection advised to take the medication(s) indicated above:**   *This will appear for each prescribed medication. | - Regularly at scheduled intervals (i.e., around the clock) - As needed (pro re nata*,* PRN) |
| 1. **Please provide further information about how you prescribe opioid medications after open colorectal resection:**   *If the medication is pre-packed, please select ‘pre-packed/determined at pharmacy’ or provide the number of pills that are usually included in a package.  * If you prescribe medications that combine two different drugs in the same pill (e.g., acetaminophen + codeine, acetaminophen + oxycodone), please indicate the number of pills of the combined medication.  *This will appear for each opioid chosen, the question will mention in a table format the name of the opioids accordingly. | \|  \| Dose per pill (in milligrams) \| Total number of pills prescribed \| \| --- \| --- \| --- \| \| Opioid 1 \| Open ended \| Drop down menu \| \| Opioid 2 \| Open ended \| Drop down menu \| |
| 1. **At hospital discharge, your prescription of analgesic medications:** | - Is the **same** for patients undergoing open or minimally invasive surgery - Is **different** between patients undergoing open or minimally invasive surgery |
| 1. **Which analgesic medications do you usually prescribe for minimally invasive colorectal resection at hospital discharge?**   *Please select ALL that apply.  * If you prescribe medications that combine two different drugs in the same pill (e.g., acetaminophen + codeine, acetaminophen + oxycodone), please select all the drugs included in the combination.  *****This will appear based on respondents’ choices whether performing minimally invasive or both (open and minimally invasive), and whether analgesia strategy is similar or not. | - Acetaminophen/Paracetamol - Ibuprofen - Naproxen - Diclofenac - Ketorolac - Celecoxib - Dipyrone/Metamizole - Gabapentinoids - Nefopam - Codeine - Hydrocodone - Nalbuphine - Hydromorphone - Morphine - Meperidine - Oxycodone (regular, immediate release) - Oxycodone (extended, slow release) - Tapentadol - Tramadol - Methadone - Transdermal opioids (e.g., fentanyl/morphine) - Other, please specify: __________________ - Analgesic medications usually not prescribed at discharge (for example, due to long hospital stay) |
| 1. **How are patients having minimally invasive resection advised to take the medication(s) indicated above:**   *This will appear for each prescribed medication. | - Regularly at scheduled intervals (i.e., around the clock) - As needed (pro re nata*,* PRN) |
| 1. **Please provide further information about how you prescribe opioid medications after minimally invasive colorectal resection:**   *If the medication is pre-packed, please select ‘pre-packed/determined at pharmacy’ or provide the number of pills that are usually included in a package.  *If you prescribe medications that combine two different drugs in the same pill (e.g., acetaminophen + codeine, acetaminophen + oxycodone), please indicate the number of pills of the combined medication.  *This will appear for each opioid chosen, the question will mention in a table format the name of the opioids accordingly. | \|  \| Dose per pill (in milligrams) \| Total number of pills prescribed \| \| --- \| --- \| --- \| \| Opioid 1 \| Open ended \| Drop down menu \| \| Opioid 2 \| Open ended \| Drop down menu \| |
| 1. **Do you usually prescribe/recommend non-pharmacological analgesia interventions to be used after hospital discharge?**   * Non-pharmacological interventions refer to pain treatments that do not involve the use of medications (e.g., ice packs, acupuncture, relaxation). | - No - Yes |
| 1. **Please specify which non-pharmacological analgesia intervention(s) you usually prescribe/recommend after hospital discharge:**   *Please select ALL that apply.  *This will appear if Yes is clicked in question 26. | - Acupuncture - Cold therapy (e.g., ice packs) - Deep breathing - Distraction (watching TV, reading) - Hypnosis - Imagery or visualization - Localized heat - Massage - Meditation - Music - Physical activity - Relaxation - Talking to friends or relatives or medical staff - TENS (Transcutaneous Electrical Nerve Stimulation) - Other, please specify: __________________ |
| 1. **Are there any other factors that typically influence your analgesia prescription at hospital discharge? [e.g., patient characteristics, preoperative diagnosis, type of resection, use of robotic surgery, type of incision, stoma creation]** | - No - Yes |
| 1. **Please specify what factor(s) typically influence your analgesia prescription at hospital discharge:**   *If Yes is clicked in question 28. |  |
| 1. **Please describe the analgesia regimen typically prescribed for the conditions indicated above:**   *If Yes is clicked in question 28. |  |
| 1. **[SURGICAL CAPTCHA VERIFICATION] To validate your responses, please indicate true or false:** | Low anterior resection is done to treat cecal cancer: True False  Part of the rectum lies underneath the peritoneal reflection: True False |
| 1. **Please provide any further comments in the box below:** |  |

Thank you for your interest in this survey. We greatly appreciate your participation!

**Please share our survey link (**[**https://mcgill.ca/x/ov4**](https://mcgill.ca/x/ov4)**) with fellow surgeons and/or trainees who perform colorectal surgeries in their practice.**

# Figure S1. X platform content used for survey distribution

**
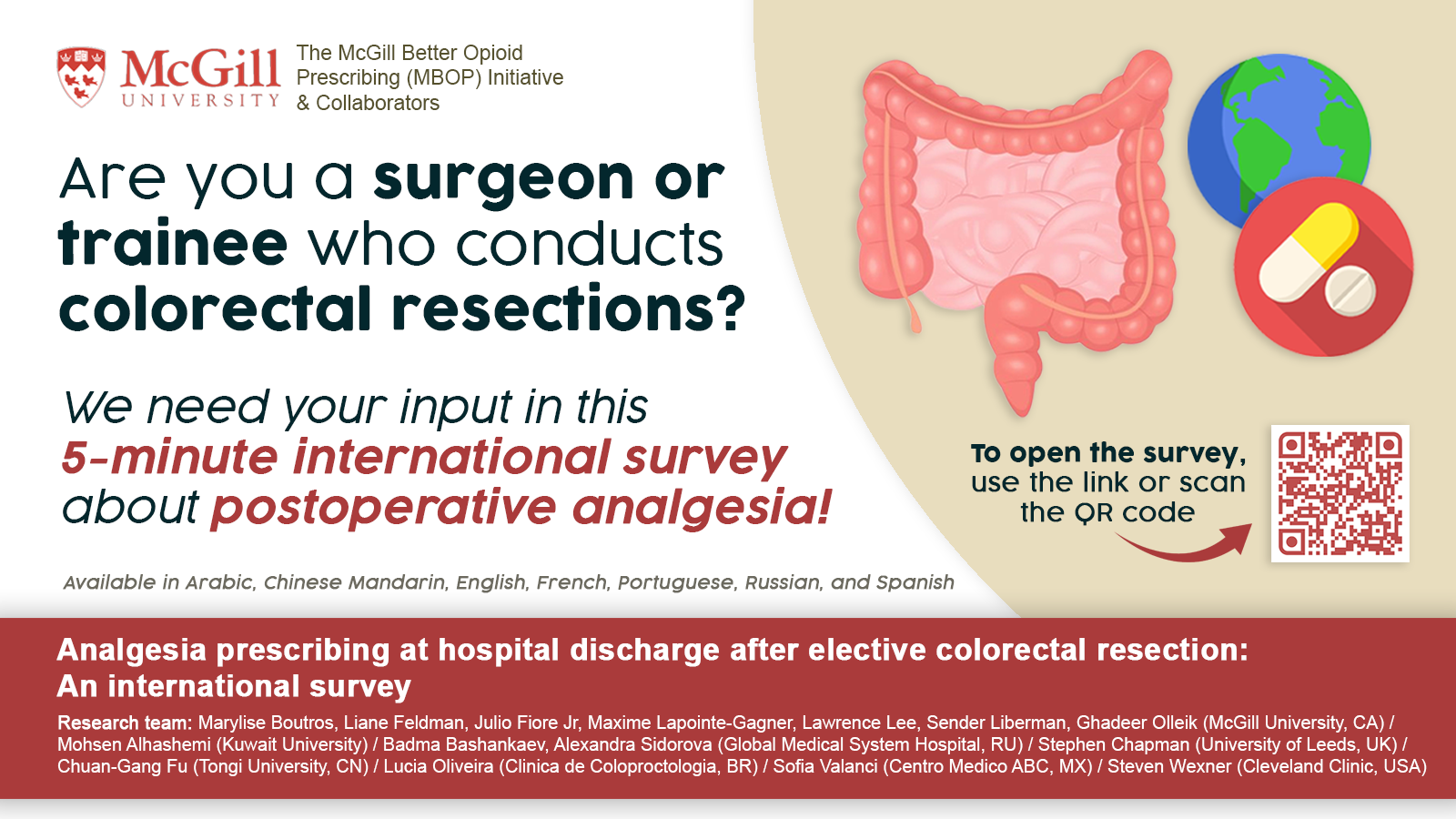
**

# Figure S2. Participant flowchart

**Survey site visitors**

**(n= 1225)**

**Survey participants**

**(n= 1025)**

**Participants included in the analysis**

**(n= 817)**

- **Visitors who did not respond any survey question (n=200)**

**Met exclusion criteria (n=208)**

- Participants who do not perform colorectal resections (n=39)
- Incomplete survey (n=107)
- Incorrect ‘captcha’ answers (n = 62)

| Table S4. Post-discharge opioid prescribing practices across different continental region, subregion, and countries (n=817) | | | | |
| --- | --- | --- | --- | --- |
|  | **n (%)** | **Overall opioid prescribing (%)** | **Opioid prescribing after open surgery (%)** | **Opioid prescribing after MIS (%)** |
| **Americas** | 423 (52%) | 313 (74%) | 219 (73%) | 288 (71%) |
| Northern America | 308 (38%) | 283 (92%) | 195 (92%) | 270 (89%) |
| *United States* | 201 (25%) | 187 (93%) | 144 (94%) | 180 (90%) |
| *Canada* | 107 (13%) | 96 (90%) | 51 (88%) | 90 (87%) |
| Latin America and the Caribbean | 115 (14%) | 30 (26%) | 24 (27%) | 18 (18%) |
| *Brazil* | 39 (5%) | 15 (38%) | 10 (37%) | 8 (25%) |
| *Mexico* | 22 (3%) | 5 (23%) | 5 (24%) | 3 (16%) |
| *Argentina* | 19 (2%) | 4 (21%) | 3 (21%) | 3 (18%) |
| *Colombia* | 19 (2%) | 3 (16%) | 3 (23%) | 1 (6%) |
| *Bolivia* | 1 (0.1%) | 0 (0%) | 0 (0%) | 0 (0%) |
| *Chile* | 5 (1%) | 1 (20%) | 1 (33%) | 1 (20%) |
| *Ecuador* | 1 (0.1%) | 0 (0%) | 0 (0%) | 0 (0%) |
| *El Salvador* | 1 (0.1%) | 0 (0%) | 0 (0%) | 0 (0%) |
| *Guatemala* | 1 (0.1%) | 0 (0%) | 0 (0%) | 0 (0%) |
| *Jamaica* | 1 (0.1%) | 1 (100%) | 1 (100%) | 1 (100%) |
| *Peru* | 2 (0.2%) | 0 (0%) | 0 (0%) | 0 (0%) |
| *Venezuela* | 3 (0.4%) | 1 (33%) | 1 (33%) | 1 (50%) |
| *Paraguay* | 1 (0.1%) | 0 (0%) | 0 (0%) | 0 (0%) |
| **Europe** | 175 (21%) | 56 (32%) | 48 (33%) | 46 (28%) |
| Northern Europe | 44 (5%) | 30 (68%) | 28 (68%) | 25 (64%) |
| *United Kingdom* | 27 (3%) | 21 (78%) | 19 (79%) | 18 (72%) |
| *Finland* | 2 (0.2%) | 2 (100%) | 2 (100%) | 0 (0%) |
| *Ireland* | 4 (0.5%) | 3 (75%) | 3 (75%) | 3 (100%) |
| *Lithuania* | 6 (1%) | 0 (0%) | 0 (0%) | 0 (0%) |
| *Sweden* | 5 (1%) | 4 (80%) | 4 (80%) | 4 (80%) |
| Table S4. Post-discharge opioid prescribing practices across different continental region, subregion, and countries (n=817) (continued) | | | | |
|  | **n (%)** | **Overall opioid prescribing (%)** | **Opioid prescribing after open surgery (%)** | **Opioid prescribing after MIS (%)** |
| Eastern Europe | 56 (7%) | 6 (11%) | 3 (7%) | 4 (8%) |
| *Russia* | 42 (5%) | 6 (14%) | 3 (9%) | 4 (11%) |
| *Belarus* | 2 (0.2%) | 0 (0%) | 0 (0%) | 0 (0%) |
| *Bulgaria* | 4 (0.5%) | 0 (0%) | 0 (0%) | 0 (0%) |
| *Hungary* | 2 (0.2%) | 0 (0%) | 0 (0%) | 0 (0%) |
| *Romania* | 2 (0.2%) | 0 (0%) | 0 (0%) | 0 (0%) |
| *Ukraine* | 4 (0.5%) | 0 (0%) | 0 (0%) | 0 (0%) |
| Southern Europe | 58 (7%) | 11 (19%) | 10 (20%) | 8 (14%) |
| *Spain* | 24 (3%) | 2 (8%) | 2 (10%) | 2 (8%) |
| *Italy* | 19 (2%) | 3 (16%) | 2 (13%) | 3 (16%) |
| *Greece* | 4 (0.5%) | 3 (75%) | 3 (75%) | 2 (67%) |
| *Portugal* | 8 (1%) | 3 (38%) | 3 (38%) | 1 (13%) |
| *Serbia* | 3 (0.4%) | 0 (0%) | 0 (0%) | 0 (0%) |
| Western Europe | 17 (2%) | 9 (53%) | 7 (64%) | 9 (53%) |
| *Austria* | 2 (0.2%) | 0 (0%) | 0 (0%) | 0 (0%) |
| *Belgium* | 1 (0.1%) | 1 (100%) | 1 (100%) | 1 (100%) |
| *France* | 4 (0.5%) | 4 (100%) | 4 (100%) | 4 (100%) |
| *Germany* | 5 (1%) | 1 (20%) | 1 (33%) | 1 (20%) |
| *Luxembourg* | 1 (0.1%) | 0 (0%) | 0 (0%) | 0 (0%) |
| *Netherlands* | 2 (0.2%) | 2 (100%) | N/A | 2 (100%) |
| *Switzerland* | 2 (0.2%) | 1 (50%) | 1 (100%) | 1 (50%) |
| **Asia** | 162 (20%) | 54 (33%) | 48 (34%) | 44 (29%) |
| Eastern Asia | 96 (12%) | 21 (22%) | 17 (21%) | 19 (20%) |
| *China* | 76 (9%) | 19 (25%) | 15 (23%) | 17 (23%) |
| *Japan* | 16 (2%) | 0 (0%) | 0 (0%) | 0 (0%) |
| Table S4. Post-discharge opioid prescribing practices across different continental region, subregion, and countries (n=817) (continued) | | | | |
|  | **n (%)** | **Overall opioid prescribing (%)** | **Opioid prescribing after open surgery (%)** | **Opioid prescribing after MIS (%)** |
| *South Korea* | 4 (1%) | 2 (50%) | 2 (50%) | 2 (50%) |
| Western Asia | 52 (6%) | 24 (46%) | 22 (47%) | 20 (43%) |
| *Kuwait* | 12 (1%) | 6 (50%) | 4 (24%) | 5 (50%) |
| *Turkey* | 24 (3%) | 8 (33%) | 8 (33%) | 7 (32%) |
| *Azerbaijan* | 1 (0.1%) | 0 (0%) | 0 (0%) | 0 (0%) |
| *Cyprus* | 1 (0.1%) | 0 (0%) | 0 (0%) | 0 (0%) |
| *Israel* | 2 (0.2%) | 1 (50%) | 1 (50%) | 1 (50%) |
| *Lebanon* | 1 (0.1%) | 0 (0%) | 0 (0%) | 0 (0%) |
| *Oman* | 1 (0.1%) | 0 (0%) | 0 (0%) | 0 (0%) |
| *Saudi Arabia* | 8 (1%) | 7 (88%) | 7 (88%) | 6 (75%) |
| *United Arab Emirates* | 1 (0.1%) | 1 (100%) | 1 (100%) | 1 (100%) |
| *Yemen* | 1 (0.1%) | 1 (100%) | 1 (100%) | N/A |
| South-eastern Asia: Malysia | 7 (1%) | 5 (71%) | 5 (71%) | 3 (43%) |
| Southern Asia | 7 (1%) | 4 (57%) | 4 (57%) | 2(40%) |
| *India* | 4 (0.5%) | 2 (50%) | 2 (50%) | 1 (33%) |
| *Bangladesh* | 2 (0.2%) | 2 (100%) | 1 (50%) | 0 (0%) |
| *Pakistan* | 1 (0.1%) | 1 (100%) | 1 (100%) | 1 (100%) |
| **Oceania** | 32 (4%) | 32 (100%) | 27 (100%) | 28 (90%) |
| Australia and New Zealand | 32 (4%) | 32 (100%) | 27 (100%) | 28 (90%) |
| *Australia* | 23 (3%) | 23 (100%) | 18 (100%) | 21 (91%) |
| *New Zealand* | 9 (1%) | 9 (100%) | 9 (100%) | 7 (88%) |
| **Africa** | 25 (3%) | 11 (44%) | 11 (44%) | 3 (23%) |
| Sub-Saharan Africa | 18 (2%) | 11 (61%) | 11 (61%) | 3(50%) |
| *Comoros* | 1 (0.1%) | 0 (0%) | 0 (0%) | 0 (0%) |
| *Ethiopia* | 8 (1%) | 6 (75%) | 6 (75%) | N/A |
| Table S4. Post-discharge opioid prescribing practices across different continental region, subregion, and countries (n=817) (continued) | | | | |
|  | **n (%)** | **Overall opioid prescribing (%)** | **Opioid prescribing after open surgery (%)** | **Opioid prescribing after MIS (%)** |
| *Kenya* | 1 (0.1%) | 1 (100%) | 1 (100%) | 0 (0%) |
| *Nigeria* | 2 (0.2%) | 1 (50%) | 1 (50%) | N/A |
| *Rwanda* | 1 (0.1%) | 0 (0%) | 0 (0%) | 0 (0%) |
| *South Africa* | 4 (0.5%) | 3 (75%) | 3 (75%) | 3 (75%) |
| *Uganda* | 1 (0.1%) | 0 (0%) | 0 (0%) | 0 (0%) |
| Northern Africa | 7 (1%) | 0 (0%) | 0 (0%) | 0 (0%) |
| *Egypt* | 5 (1%) | 0 (0%) | 0 (0%) | 0 (0%) |
| *Libya* | 1 (0.1%) | 0 (0%) | 0 (0%) | 0 (0%) |
| *Sudan* | 1 (0.1%) | 0 (0%) | 0 (0%) | 0 (0%) |
| **Data are reported as n (%).**  Number of responders is less than the cumulative frequency of open and minimally invasive surgery due to multiple choice answers.  MIS: minimally invasive surgery. | | | | |

| Table S5. In-hospital care characteristics in different world subregions | | | | | | | | | | | | | | |
| --- | --- | --- | --- | --- | --- | --- | --- | --- | --- | --- | --- | --- | --- | --- |
|  | Overall | Northern America | Latin America and the Caribbean | Northern Europe | Eastern Europe | Southern Europe | Western Europe | Eastern Asia | Western Asia | South-eastern Asia | Southern Asia | Australia and New Zealand | Sub-Saharan Africa | Northern Africa |
| **Use of enhanced recovery pathway** | 639/817 (78%) | 283/308 (92%) | 51/115 (44%) | 40/44 (91%) | 47/56 (84%) | 42/58 (72%) | 14/17 (82%) | 83/96 (86%) | 33/52 (63%) | 5/7 (71%) | 5/7 (71%) | 28/32 (88%) | 3/18 (17%) | 5/7 (71%) |
| *Open Surgery (n=640)* | 494/640 (77%) | 197/211 (93%) | 39/89 (44%) | 37/41 (90%) | 35/44 (80%) | 36/51 (71%) | 9/11 (82%) | 70/80 (88%) | 30/47 (64%) | 5/7 (71%) | 5/7 (71%) | 23/27 (85%) | 3/18 (17%) | 5/7 (71%) |
| *MIS (n=760)* | 617/760 (81%) | 281/305 (92%) | 43/98 (44%) | 38/39 (97%) | 44/50 (88%) | 42/56 (75%) | 14/17 (82%) | 80/93 (86%) | 32/46 (70%) | 5/7 (71%) | 4/5 (80%) | 27/31 (87%) | 2/6 (33%) | 5/7 (71%) |
| **Length of stay, median (IQR)** |  |  |  |  |  |  |  |  |  |  |  |  |  |  |
| *Open Surgery (n=640)* | 5 (5–7) | 5 (4–5) | 5 (4–6) | 6 (5–7) | 8 (7-10) | 6 (5–7) | 7 (7–9) | 10 (8-13) | 5 (5–7) | 6 (5–7) | 7 (6–8) | 7 (6–7) | 7 (5–9) | 6 (4–7) |
| *MIS (n=760)* | 4 (3–5) | 3 (2–3) | 4 (3–4) | 4 (3–5) | 6 (5-7) | 5 (3–7) | 5 (4–6) | 7 (7– 10) | 5 (4–5) | 4 (3–5) | 4 (4–6) | 4 (4–5) | 6 (4–7) | 5 (4–6) |
| **Methods of in-hospital analgesia** |  |  |  |  |  |  |  |  |  |  |  |  |  |  |
| Peripheral nerve block | 329/817 (40%) | 188/308 (61%) | 15/115 (13%) | 25/45  (56%) | 7/56  (13%) | 27/58  (47%) | 3/17  (18%) | 24/96 (25%) | 7/52  (13%) | 2/7  (29%) | 2/7  (29%) | 24/32 (75%) | 3/18  (17%) | 2/7  (29%) |
| *Open Surgery (n=640)* | 243/640 (38%) | 131/211 (62%) | 7/89  (8%) | 24/41 (59%) | 4/44  (9%) | 22/51 (43%) | 2/11  (18%) | 18/80 (23%) | 5/47  (11%) | 2/7  (29%) | 2/7  (29%) | 22/27 (81%) | 3/18  (17%) | 1/7  (14%) |
| *MIS (n=760)* | 294/760 (39%) | 178/305 (58%) | 13/98 (13%) | 19/39 (49%) | 5/50  (10%) | 23/56 (41%) | 2/17  (12%) | 19/93 (20%) | 7/46  (15%) | 1/7  (14%) | 1/5  (20%) | 21/31 (68%) | 3/6  (50%) | 2/7  (29%) |
| Table S5. In-hospital care characteristics in different world subregions (continued) | | | | | | | | | | | | | | |
|  | Overall | Northern America | Latin America and the Caribbean | Northern Europe | Eastern Europe | Southern Europe | Western Europe | Eastern Asia | Western Asia | South-eastern Asia | Southern Asia | Australia and New Zealand | Sub-Saharan Africa | Northern Africa |
| Wound infiltration | 324/817 (40%) | 166/308  (54%) | 34/115  (30%) | 24/44 (55%) | 4/56  (7%) | 28/58 (48%) | 4/17  (24%) | 20/96  (21%) | 10/52 (19%) | 7/7  (100%) | 3/7 (43%) | 17/32 (53%) | 4/18  (22%) | 3/7  (43%) |
| *Open Surgery (n=640)* | 180/640 (28%) | 67/211 (32%) | 24/89 (27%) | 13/41 (32%) | 2/44  (5%) | 23/51 (45%) | 2/11  (18%) | 14/80 (18%) | 9/47  (19%) | 5/7  (71%) | 2/7  (25%) | 12/27 (44%) | 4/18  (22%) | 3/7  (43%) |
| *MIS (n=760)* | 301/760 (40%) | 164/305 (54%) | 29/98 (30%) | 23/39 (59%) | 3/50  (6%) | 24/56 (43%) | 4/17  (24%) | 16/93 (17%) | 10/46 (22%) | 6/7  (86%) | 1/5  (20%) | 17/31 (55%) | 1/6  (17%) | 3/7  (43%) |
| Epidural analgesia | 296/817 (36%) | 95/308 (31%) | 36/115 (31%) | 19/44 (43%) | 42/56 (75%) | 36/58 (62%) | 9/17  (53%) | 21/96 (22%) | 18/52 (35%) | 4/7  (57%) | 4/7  (57%) | 6/32  (19%) | 3/18  (17%) | 3/7  (43%) |
| *Open Surgery (n=640)* | 259/640 (40%) | 78/211 (37%) | 31/89 (35%) | 19/41 (46%) | 33/44 (75%) | 34/51 (67%) | 7/11 (64%) | 19/80 (24%) | 18/47 (38%) | 4/7  (57%) | 4/7  (57%) | 6/27  (22%) | 3/18  (17%) | 3/7  (43%) |
| *MIS (n=760)* | 175/760 (23%) | 50/305 (16%) | 27/98 (28%) | 7/39  (18%) | 31/50 (62%) | 17/56 (30%) | 5/17  (29%) | 14/93 (15%) | 13/46 (28%) | 2/7  (29%) | 1/5  (20%) | 3/31  (10%) | 2/6  (33%) | 3/7  (43%) |
| Spinal analgesia | 95/817 (12%) | 23/308  (7%) | 13/115 (11%) | 22/44 (50%) | 6/56  (11%) | 8/58  (14%) | 3/17  (18%) | 3/96  (3%) | 1/52  (2%) | 0/7  (0%) | 0/7  (0%) | 13/32 (41%) | 3/18  (17%) | 0/7  (0%) |
| *Open Surgery (n=640)* | 74/640 (12%) | 14/211  (7%) | 13/89 (15%) | 15/41 (37%) | 5/44  (11%) | 6/51  (12%) | 3/11  (27%) | 2/80  (3%) | 1/47  (2%) | 0/7  (0%) | 0/7  (0%) | 12/27 (44%) | 3/18  (17%) | 0/7  (0%) |
| *MIS (n=760)* | 79/760 (10%) | 20/305  (7%) | 9/98  (9%) | 21/39 (54%) | 4/50  (8%) | 6/56  (11%) | 2/17  (12%) | 2/93  (2%) | 1/46  (2%) | 0/7  (0%) | 0/5  (0%) | 11/31 (35%) | 3/6  (50%) | 0/7  (0%) |
| Intravenous analgesia (inc. PCA) | 625/817 (77%) | 197/308 (64%) | 97/115 (84%) | 36/44 (82%) | 39/56 (70%) | 50/58 (86%) | 16/17 (94%) | 82/96 (85%) | 46/52 (88%) | 7/7 (100%) | 5/7  (71%) | 31/32 (97%) | 14/18 (78%) | 5/7  (71%) |
| *Open Surgery (n=640)* | 515/640 (80%) | 157/211 (74%) | 74/89 (83%) | 33/41 (80%) | 30/44 (68%) | 46/51 (90%) | 11/11 (100%) | 66/80 (83%) | 40/47 (85%) | 7/7 (100%) | 5/7  (71%) | 27/27 (100%) | 14/18 (78%) | 5/7  (71%) |
| Table S5. In-hospital care characteristics in different world subregions (continued) | | | | | | | | | | | | | | |
|  | Overall | Northern America | Latin America and the Caribbean | Northern Europe | Eastern Europe | Southern Europe | Western Europe | Eastern Asia | Western Asia | South-eastern Asia | Southern Asia | Australia and New Zealand | Sub-Saharan Africa | Northern Africa |
| *MIS (n=760)* | 522/760 (69%) | 167/305 (55%) | 80/98 (82%) | 29/39 (74%) | 31/50 (62%) | 45/56 (80%) | 15/17 (88%) | 76/93 (82%) | 40/46 (87%) | 5/7  (71%) | 4/5  (80%) | 24/31 (77%) | 3/6  (50%) | 5/7 (71%) |
| Oral analgesia | 569/817 (70%) | 274/308 (89%) | 59/115 (51%) | 38/44 (86%) | 29/56 (52%) | 34/58 (59%) | 15/17 (88%) | 40/96 (58%) | 21/52 (40%) | 7/7 (100%) | 6/7  (86%) | 32/32 (100%) | 11/18 (61%) | 3/7  (43%) |
| *Open Surgery (n=640)* | 425/640 (66%) | 180/211 (85%) | 42/89 (47%) | 36/41 (88%) | 23/44 (52%) | 27/51 (53%) | 11/11 (100%) | 32/80 (40%) | 20/47 (43%) | 7/7 (100%) | 6/7  (86%) | 27/27 (100%) | 11/18 (61%) | 3/7 (43%) |
| *MIS (n=760)* | 524/760 (69%) | 271/305 (89%) | 48/98 (49%) | 33/39 (85%) | 26/50 (52%) | 30/56 (54%) | 15/17 (88%) | 35/93 (38%) | 20/46 (43%) | 6/7  (86%) | 3/5  (60%) | 31/31 (100%) | 3/6  (50%) | 3/7  (43%) |
| Rectal analgesia | 20/817 (2%) | 8/308  (3%) | 0/115  (0%) | 1/44  (2%) | 0/56  (0%) | 1/58  (2%) | 0/17  (0%) | 6/96  (6%) | 1/52  (2%) | 1/7  (14%) | 0/7  (0%) | 0/32  (0%) | 2/18  (11%) | 0/7  (0%) |
| *Open Surgery (n=640)* | 13/640 (2%) | 1/211  (0%) | 0/89  (0%) | 1/41  (2%) | 0/44  (0%) | 1/51  (2%) | 0/11  (0%) | 6/80  (8%) | 1/47  (2%) | 1/7  (14%) | 0/7  (0%) | 0/27  (0%) | 2/18  (11%) | 0/7  (0%) |
| *MIS (n=760)* | 13/760 (2%) | 7/305  (2%) | 0/98  (0%) | 0/39  (0%) | 0/50  (0%) | 0/56  (0%) | 0/17  (0%) | 4/93  (4%) | 1/46  (2%) | 1/7  (14%) | 0/5  (0%) | 0/31  (0%) | 0/6  (0%) | 0/7  (0%) |
| **Analgesic drugs/classes used in-hospital** |  |  |  |  |  |  |  |  |  |  |  |  |  |  |
| Opioids | 614/817 (75%) | 290/308 (94%) | 64/115 (56%) | 38/44 (86%) | 22/56 (39%) | 26/58 (45%) | 14/17 (82%) | 51/96 (53%) | 43/52 (83%) | 6/7  (86%) | 6/7  (86%) | 32/32 (100%) | 16/18 (89%) | 6/7  (86%) |
| *Open Surgery (n=640)* | 487/640 (76%) | 202/211 (96%) | 50/89 (56%) | 36/41 (88%) | 21/44 (48%) | 23/51 (45%) | 11/11 (100%) | 42/80 (53%) | 41/47 (87%) | 6/7  (86%) | 6/7  (86%) | 27/27 (100%) | 16/18 (89%) | 6/7  (86%) |
| *MIS (n=760)* | 525/760 (69%) | 275/305 (90%) | 48/98 (49%) | 34/39 (87%) | 13/50 (26%) | 16/56 (29%) | 13/17 (76%) | 43/93 (46%) | 36/46 (78%) | 3/7  (43%) | 4/5  (80%) | 30/31 (97%) | 5/6  (83%) | 5/7  (71%) |
| Table S5. In-hospital care characteristics in different world subregions (continued) | | | | | | | | | | | | | | |
|  | Overall | Northern America | Latin America and the Caribbean | Northern Europe | Eastern Europe | Southern Europe | Western Europe | Eastern Asia | Western Asia | South-eastern Asia | Southern Asia | Australia and New Zealand | Sub-Saharan Africa | Northern Africa |
| Traditional local anesthetics | 405/817 (50%) | 210/308 (68%) | 29/115 (25%) | 33/44 (75%) | 17/56 (30%) | 36/58 (62%) | 10/17 (59%) | 20/96 (21%) | 10/52 (19%) | 6/7  (86%) | 4/7  (57%) | 24/32 (75%) | 4/18  (22%) | 2/7  (29%) |
| *Open Surgery (n=640)* | 297/640 (46%) | 139/211 (66%) | 17/89 (19%) | 30/41 (73%) | 13/44 (30%) | 30/51 (59%) | 8/11  (73%) | 16/80 (20%) | 10/47 (21%) | 6/7 (86%) | 3/7  (43%) | 20/27 (74%) | 3/18  (17%) | 2/7  (29%) |
| *MIS (n=760)* | 364/760 (48%) | 198/305 (65%) | 25/98 (26%) | 31/39 (79%) | 12/50 (24%) | 31/56 (55%) | 7/17  (41%) | 17/93 (18%) | 10/46 (22%) | 3/7  (43%) | 2/5  (40%) | 23/31 (74%) | 3/6  (50%) | 2/7  (29%) |
| Sustained release local anesthetics | 125/817 (15%) | 74/308 (24%) | 13/115 (11%) | 6/44  (14%) | 3/56  (5%) | 9/58  (16%) | 1/17  (6%) | 8/96  (8%) | 2/52  (4%) | 2/7  (29%) | 0/7  (0%) | 6/32  (19%) | 1/18  (6%) | 0/7  (0%) |
| *Open Surgery (n=640)* | 94/640 (15%) | 53/211 (25%) | 12/89 (13%) | 5/41  (12%) | 1/44  (2%) | 9/51  (18%) | 1/11  (9%) | 2/80  (3%) | 2/47  (4%) | 2/7  (29%) | 0/7  (0%) | 6/27  (22%) | 1/18  (6%) | 0/7  (0%) |
| *MIS (n=760)* | 112/760 (15%) | 71/305 (23%) | 9/98 (9%) | 5/39  (13%) | 3/50  (6%) | 9/56  (16%) | 1/17  (6%) | 7/93  (8%) | 1/46  (2%) | 2/7  (29%) | 0/5  (0%) | 3/31  (10%) | 1/6  (17%) | 0/7  (0%) |
| Acetaminophen/Paracetamol, intravenous | 371/817 (45%) | 94/308 (31%) | 45/115 (39%) | 32/44 (73%) | 25/56 (45%) | 48/58 (83%) | 9/17  (53%) | 26/96 (27%) | 43/52 (83%) | 5/7  (71%) | 6/7  (86%) | 25/32 (78%) | 8/18  (44%) | 5/7  (71%) |
| *Open Surgery (n=640)* | 314/640 (49%) | 77/211 (36%) | 36/89 (40%) | 31/41 (76%) | 17/44 (39%) | 41/51 (80%) | 7/11  (64%) | 19/80 (24%) | 40/47 (85%) | 5/7 (71%) | 6/8  (86%) | 22/27 (81%) | 8/18  (44%) | 5/7  (71%) |
| *MIS (n=760)* | 334/760 (44%) | 90/305 (30%) | 40/98 (41%) | 27/39 (69%) | 18/50 (36%) | 46/56 (82%) | 7/17  (41%) | 24/93 (26%) | 39/46 (85%) | 5/7  (71%) | 4/5  (80%) | 24/31 (77%) | 5/6  (83%) | 5/7  (71%) |
| Acetaminophen/Paracetamol, oral | 449/817 (55%) | 274/308 (89%) | 25/115 (22%) | 31/44 (70%) | 8/56  (14%) | 29/58 (50%) | 8/17  (47%) | 16/96 (17%) | 16/52 (32%) | 4/7  (57%) | 4/7  (57%) | 29/32 (91%) | 4/18  (22%) | 1/7  (14%) |
| *Open Surgery (n=640)* | 329/640 (51%) | 181/211 (86%) | 18/89 (20%) | 29/41 (71%) | 6/44  (14%) | 24/51 (47%) | 6/11  (55%) | 13/80 (16%) | 15/47 (32%) | 4/7  (57%) | 4/7  (57%) | 25/27 (93%) | 3/18  (17%) | 1/7  (14%) |
| Table S5. In-hospital care characteristics in different world subregions (continued) | | | | | | | | | | | | | | |
|  | Overall | Northern America | Latin America and the Caribbean | Northern Europe | Eastern Europe | Southern Europe | Western Europe | Eastern Asia | Western Asia | South-eastern Asia | Southern Asia | Australia and New Zealand | Sub-Saharan Africa | Northern Africa |
| *MIS (n=760)* | 424/760 (56%) | 269/305 (88%) | 21/98 (21%) | 29/39 (74%) | 7/50  (14%) | 26/56 (46%) | 8/17  (47%) | 13/93 (14%) | 15/46 (34%) | 4/7  (57%) | 2/5  (40%) | 27/31 (87%) | 2/6  (33%) | 1/7 (14%) |
| NSAIDs | 554/817 (68%) | 216/308 (70%) | 73/115 (63%) | 27/44 (61%) | 52/56 (93%) | 40/58 (69%) | 7/17  (41%) | 59/96 (61%) | 34/52 (65%) | 6/7  (86%) | 5/8  (71%) | 15/32 (47%) | 15/18 (83%) | 5/7  (71%) |
| *Open Surgery (n=640)* | 435/640 (68%) | 153/211 (73%) | 59/89 (66%) | 23/41 (56%) | 40/44 (91%) | 35/51 (69%) | 5/11  (45%) | 48/80 (60%) | 29/47 (62%) | 6/7  (86%) | 5/7  (71%) | 12/27 (44%) | 15/18 (83%) | 5/7  (71%) |
| *MIS (n=760)* | 510/760 (67%) | 214/305 (70%) | 61/98 (62%) | 23/39 (59%) | 47/50 (94%) | 39/56 (70%) | 7/17  (41%) | 53/93 (57%) | 31/46 (67%) | 6/7  (86%) | 4/5  (80%) | 15/31 (48%) | 5/6  (83%) | 5/7 (71%) |
| Dipyrone/Metamizole | 111/817 (14%) | 1/308  (0%) | 58/115 (50%) | 1/44  (2%) | 12/56 (21%) | 26/58 (45%) | 7/17  (41%) | 2/96  (2%) | 3/52  (6%) | 0/7  (0%) | 0/7  (0%) | 0/32  (0%) | 1/18  (6%) | 0/7  (0%) |
| *Open Surgery (n=640)* | 83/640 (13%) | 1/211  (0%) | 42/89 (47%) | 1/41  (2%) | 8/44  (18%) | 21/51 (41%) | 5/11  (45%) | 1/80  (1%) | 3/47  (6%) | 0/7  (0%) | 0/7  (0%) | 0/27  (0%) | 1/18  (6%) | 0/7  (0%) |
| *MIS (n=760)* | 95/760 (13%) | 1/305  (0%) | 47/98 (48%) | 1/39  (3%) | 9/50  (18%) | 24/56 (43%) | 7/17  (41%) | 2/93  (2%) | 3/46  (7%) | 0/7  (0%) | 0/5  (0%) | 0/31  (0%) | 1/6  (17%) | 0/7  (0%) |
| Gabapentinoids | 166/817 (20%) | 147/308 (48%) | 3/115  (3%) | 5/44  (11%) | 1/56  (2%) | 1/58  (2%) | 0/17  (0%) | 1/96  (1%) | 3/52  (6%) | 0/7  (0%) | 1/7  (14%) | 4/32  (13%) | 0/18  (0%) | 0/7  (0%) |
| *Open Surgery (n=640)* | 117/640 (18%) | 102/211 (48%) | 2/89  (2%) | 5/41  (12%) | 0/44  (0%) | 1/51  (2%) | 0/11  (0%) | 1/80  (1%) | 1/47  (2%) | 0/7  (0%) | 1/7  (14%) | 4/27  (15%) | 0/18  (0%) | 0/7  (0%) |
| *MIS (n=760)* | 162/760 (21%) | 145/305 (48%) | 2/98  (2%) | 5/39  (13%) | 1/50  (2%) | 1/56  (2%) | 0/17  (0%) | 1/93  (1%) | 3/46  (7%) | 0/7  (0%) | 1/5  (20%) | 3/31  (10%) | 0/6  (0%) | 0/7  (0%) |
| Table S5. In-hospital care characteristics in different world subregions (continued) | | | | | | | | | | | | | | |
|  | Overall | Northern America | Latin America and the Caribbean | Northern Europe | Eastern Europe | Southern Europe | Western Europe | Eastern Asia | Western Asia | South-eastern Asia | Southern Asia | Australia and New Zealand | Sub-Saharan Africa | Northern Africa |
| Dexmedetomidine | 10/817 (1%) | 3/308  (1%) | 1/115  (1%) | 0/44  (0%) | 2/56  (4%) | 1/58  (2%) | 0/17  (0%) | 1/96  (1%) | 0/52  (0%) | 0/7  (0%) | 1/7  (14%) | 0/32  (0%) | 1/18  (6%) | 0/7  (0%) |
| *Open Surgery (n=640)* | 8/640 (1%) | 1/211  (0%) | 1/89  (1%) | 0/41  (0%) | 2/44  (5%) | 1/51  (2%) | 0/11  (0%) | 1/80  (1%) | 0/47  (0%) | 0/7  (0%) | 1/7  (14%) | 0/27  (0%) | 1/18  (6%) | 0/7  (0%) |
| *MIS (n=760)* | 8/760 (1%) | 3/305  (1%) | 1/98  (1%) | 0/39  (0%) | 1/50  (2%) | 1/56  (2%) | 0/17 (0%) | 1/93  (1%) | 0/46  (0%) | 0/7  (0%) | 1/5  (20%) | 0/31  (0%) | 0/6  (0%) | 0/7  (0%) |
| Nefopam | 5/817 (1%) | 0/308  (0%) | 0/115  (0%) | 0/44  (0%) | 2/56  (4%) | 0/58  (0%) | 3/17  (18%) | 0/96  (0%) | 0/52  (0%) | 0/7  (0%) | 0/7  (0%) | 0/32  (0%) | 0/18  (0%) | 0/7  (0%) |
| *Open Surgery (n=640)* | 5/640 (1%) | 0/211  (0%) | 0/89  (0%) | 0/41  (0%) | 2/44  (5%) | 0/51  (0%) | 3/11  (27%) | 0/80  (0%) | 0/47  (0%) | 0/7  (0%) | 0/7  (0%) | 0/27  (0%) | 0/18  (0%) | 0/7  (0%) |
| *MIS (n=760)* | 3/760 (<1%) | 0/305  (0%) | 0/98  (0%) | 0/39  (0%) | 1/50  (2%) | 0/56  (0%) | 2/17  (12%) | 0/93  (0%) | 0/46  (0%) | 0/7  (0%) | 0/5  (0%) | 0/31  (0%) | 0/6  (0%) | 0/7  (0%) |
| Clonidine | 10/817 (1%) | 1/308  (0%) | 0/115  (0%) | 2/44  (5%) | 0/56  (0%) | 1/58  (2%) | 0/17  (0%) | 1/96  (1%) | 0/52  (0%) | 0/7  (0%) | 0/7  (0%) | 5/32  (16%) | 0/18  (0%) | 0/7  (0%) |
| *Open Surgery (n=640)* | 10/640 (2%) | 1/211  (0%) | 0/89  (0%) | 2/41  (5%) | 0/44  (0%) | 1/51  (2%) | 0/11  (0%) | 1/80  (1%) | 0/47  (0%) | 0/7  (0%) | 0/7  (0%) | 5/27  (19%) | 0/18  (0%) | 0/7  (0%) |
| *MIS (n=760)* | 4/760 (1%) | 1/305  (0%) | 0/98  (0%) | 0/39  (0%) | 0/50  (0%) | 0/56  (0%) | 0/17  (0%) | 0/93  (0%) | 0/46  (0%) | 0/7  (0%) | 0/5  (0%) | 3/31  (10%) | 0/6  (0%) | 0/7  (0%) |
| Ketamine | 31/817 (4%) | 13/308  (4%) | 1/115  (1%) | 2/44  (5%) | 2/56  (4%) | 0/58  (0%) | 1/17  (6%) | 1/96  (1%) | 1/52  (2%) | 0/7  (0%) | 0/7  (0%) | 10/32 (31%) | 0/18  (0%) | 0/7  (0%) |
| *Open Surgery (n=640)* | 23/640 (4%) | 7/211  (3%) | 1/89  (1%) | 2/41  (5%) | 2/44  (5%) | 0/51  (0%) | 0/11  (0%) | 0/80  (0%) | 1/47  (2%) | 0/7  (0%) | 0/7  (0%) | 10/27 (37%) | 0/18  (0%) | 0/7  (0%) |
| Table S5. In-hospital care characteristics in different world subregions (continued) | | | | | | | | | | | | | | |
|  | Overall | Northern America | Latin America and the Caribbean | Northern Europe | Eastern Europe | Southern Europe | Western Europe | Eastern Asia | Western Asia | South-eastern Asia | Southern Asia | Australia and New Zealand | Sub-Saharan Africa | Northern Africa |
| *MIS (n=760)* | 25/760 (3%) | 12/305  (4%) | 1/98  (1%) | 2/39  (5%) | 2/50  (4%) | 0/56  (0%) | 1/17  (6%) | 1/93  (1%) | 0/46  (0%) | 0/7  (0%) | 0/5  (0%) | 6/31  (0%) | 0/6  (0%) | 0/7  (0%) |

**Data are reported as frequency n (%) or median (IQR).**

The total numbers of participants (n=817) is less than the cumulative frequency of open surgery (n=640) and MIS (n=760) because many participants conducted both types of procedures.
NSAIDs: non-steroidal anti-inflammatory drugs, PCA: patient-controlled analgesia, MIS: minimally invasive surgery.

| Table S6. Opioid medications prescribed at discharge in different world subregions | | | | | | | | | | |  |  |  |  |  |
| --- | --- | --- | --- | --- | --- | --- | --- | --- | --- | --- | --- | --- | --- | --- | --- |
|  | Overall | Northern America | Latin America and the Caribbean | Northern Europe | Eastern Europe | Southern Europe | Western Europe | Eastern Asia | Western Asia | South-eastern Asia | | Southern Asia | Australia and New Zealand | Sub-Saharan Africa | Northern Africa |
| **Tramadol** | 198/466 (42%) | 85/283 (30%) | 22/30 (73%) | 9/30 (30%) | 5/6 (83%) | 7/11 (64%) | 6/9 (67%) | 13/21 (62%) | 20/24 (83%) | 5/5 (100%) | | 4/4 (100%) | 13/32 (41%) | 9/11 (82%) | 0/7  (0%) |
| *Open Surgery* | 157/353 (44%) | 55/195 (28%) | 20/24 (83%) | 8/28 (29%) | 2/3 (67%) | 7/10 (70%) | 5/7 (71%) | 11/17 (65%) | 18/22 (82%) | 5/5 (100%) | | 4/4 (100%) | 13/27 (48%) | 9/11 (82%) | 0/7  (0%) |
| *MIS* | 157/409 (38%) | 78/270 (29%) | 13/18 (72%) | 6/25 (24%) | 4/4 (100%) | 4/8 (50%) | 6/9 (67%) | 11/19 (58%) | 17/20 (85%) | 3/3 (100%) | | 2/2 (100%) | 11/28 (39%) | 2/3 (67%) | 0/7  (0%) |
| **Oxycodone (immediate release)** | 159/466 (34%) | 130/283 (46%) | 1/30 (3%) | 9/30 (30%) | 0/6 (0%) | 1/11 (9%) | 1/9 (11%) | 1/21 (5%) | 1/24 (4%) | 0/5 (0%) | | 0/4 (0%) | 15/32 (47%) | 0/11 (0%) | 0/7  (0%) |
| *Open Surgery* | 124/353 (35%) | 102/195 (52%) | 0/24 (0%) | 9/28 (32%) | 0/3 (0%) | 1/10 (10%) | 0/7 (0%) | 0/17 (0%) | 1/22 (5%) | 0/5 (0%) | | 0/4 (0%) | 11/27 (41%) | 0/11 (0%) | 0/7  (0%) |
| *MIS* | 141/409 (34%) | 116/270 (43%) | 1/18 (6%) | 8/25 (32%) | 0/4 (0%) | 1/8 (13%) | 1/9 (11%) | 1/19 (5%) | 1/20 (5%) | 0/3 (0%) | | 0/2 (0%) | 12/28 (43%) | 0/3 (0%) | 0/7  (0%) |
| **Hydromorphone** | 68/466 (15%) | 68/283 (24%) | 0/30 (0%) | 0/30 (0%) | 0/6 (0%) | 0/11 (0%) | 0/9 (0%) | 0/21 (0%) | 0/24 (0%) | 0/5 (0%) | | 0/4 (0%) | 0/32 (0%) | 0/11 (0%) | 0/7  (0%) |
| *Open Surgery* | 34/353 (10%) | 34/195 (17%) | 0/24 (0%) | 0/28 (0%) | 0/3 (0%) | 0/10 (0%) | 0/7 (0%) | 0/17 (0%) | 0/22 (0%) | 0/5 (0%) | | 0/4 (0%) | 0/27 (0%) | 0/11 (0%) | 0/7  (0%) |
| *MIS* | 64/409 (16%) | 64/270 (24%) | 0/18 (0%) | 0/25 (0%) | 0/4 (0%) | 0/8 (0%) | 0/9 (0%) | 0/19 (0%) | 0/20 (0%) | 0/3 (0%) | | 0/2 (0%) | 0/28 (0%) | 0/3 (0%) | 0/7  (0%) |
| **Codeine** | 36/466 (8%) | 7/283 (2%) | 7/30 (23%) | 15/30 (50%) | 0/6 (0%) | 2/11 (18%) | 1/9 (11%) | 0/21 (0%) | 2/24 (8%) | 0/5 (0%) | | 0/4 (0%) | 1/32 (3%) | 1/11 (9%) | 0/7  (0%) |
| Table S6. Opioid medications prescribed at discharge in different world subregions (continued) | | | | | | | | | | | | | | | |
|  | Overall | Northern America | Latin America and the Caribbean | Northern Europe | Eastern Europe | Southern Europe | Western Europe | Eastern Asia | Western Asia | South-eastern Asia | | Southern Asia | Australia and New Zealand | Sub-Saharan Africa | Northern Africa |
| *Open Surgery* | 32/353 (9%) | 7/195 (4%) | 4/24 (17%) | 14/28 (50%) | 0/3 (0%) | 2/10 (20%) | 1/7 (14%) | 0/17 (0%) | 2/22 (9%) | 0/5 (0%) | | 0/4 (0%) | 1/27 (4%) | 1/11 (9%) | 0/7  (0%) |
| *MIS* | 30/409  (7%) | 7/270 (3%) | 5/18 (28%) | 13/25 (52%) | 0/4 (0%) | 1/8 (13%) | 1/9 (11%) | 0/19 (0%) | 2/20 (10%) | 0/3 (0%) | | 0/2 (0%) | 1/28 (4%) | 0/3 (0%) | 0/0  (0%) |
| **Hydrocodone** | 43/466 (9%) | 40/283 (14%) | 1/30 (3%) | 0/30 (0%) | 0/6 (0%) | 1/11 (9%) | 0/9 (0%) | 0/21 (0%) | 0/24 (0%) | 0/5 (0%) | | 0/4 (0%) | 0/32 (0%) | 1/11 (9%) | 0/0  (0%) |
| *Open Surgery* | 32/353 (9%) | 29/195 (15%) | 1/24 (4%) | 0/28 (0%) | 0/3 (0%) | 1/10 (10%) | 0/7 (0%) | 0/17 (0%) | 0/22 (0%) | 0/5 (0%) | | 0/4 (0%) | 0/27 (0%) | 1/11 (9%) | 0/0  (0%) |
| *MIS* | 38/409 (9%) | 37/270 (14%) | 0/18 (0%) | 0/25 (0%) | 0/4 (0%) | 0/8 (0%) | 0/9 (0%) | 0/19 (0%) | 0/20 (0%) | 0/3 (0%) | | 0/2 (0%) | 0/28 (0%) | 1/3 (33%) | 0/0  (0%) |
| **Morphine** | 35/466 (8%) | 26/283 (9%) | 0/30 (0%) | 4/30 (13%) | 0/6 (0%) | 3/11 (27%) | 0/9 (0%) | 0/21 (0%) | 2/24 (8%) | 0/5 (0%) | | 0/4 (0%) | 0/32 (0%) | 0/11 (0%) | 0/0  (0%) |
| *Open Surgery* | 23/353 (7%) | 14/195 (7%) | 0/24 (0%) | 4/28 (14%) | 0/3 (0%) | 3/10 (30%) | 0/7 (0%) | 0/17 (0%) | 2/22 (9%) | 0/5 (0%) | | 0/4 (0%) | 0/27 (0%) | 0/11 (0%) | 0/0  (0%) |
| *MIS* | 31/409 (8%) | 24/270 (9%) | 0/18 (0%) | 4/25 (16%) | 0/4 (0%) | 2/8 (25%) | 0/9 (0%) | 0/19 (0%) | 1/20 (5%) | 0/3 (0%) | | 0/2 (0%) | 0/28 (0%) | 0/3 (0%) | 0/0  (0%) |
| **Oxycodone (slow release)** | 27/466 (6%) | 3/283 (1%) | 0/30 (0%) | 8/30 (27%) | 0/6 (0%) | 0/11 (0%) | 3/9 (33%) | 8/21 (38%) | 0/24 (0%) | 0/5 (0%) | | 0/4 (0%) | 5/32 (16%) | 0/11 (0%) | 0/0  (0%) |
| *Open Surgery* | 23/353 (7%) | 2/195 (1%) | 0/24 (0%) | 8/28 (29%) | 0/3 (0%) | 0/10 (0%) | 2/7 (29%) | 6/17 (35%) | 0/22 (0%) | 0/5 (0%) | | 0/4 (0%) | 5/27 (19%) | 0/11 (0%) | 0/0  (0%) |
| *MIS* | 23/409 (6%) | 3/270 (1%) | 0/18 (0%) | 5/25 (20%) | 0/4 (0%) | 0/8 (0%) | 3/9 (33%) | 8/19 (42%) | 0/20 (0%) | 0/3 (0%) | | 0/2 (0%) | 4/28 (14%) | 0/3 (0%) | 0/0  (0%) |
| Table S6. Opioid medications prescribed at discharge in different world subregions (continued) | | | | | | | | | | | | | | | |
|  | Overall | Northern America | Latin America and the Caribbean | Northern Europe | Eastern Europe | Southern Europe | Western Europe | Eastern Asia | Western Asia | South-eastern Asia | | Southern Asia | Australia and New Zealand | Sub-Saharan Africa | Northern Africa |
| **Tapentadol** | 16/466  (3%) | 0/283 (0%) | 0/30 (0%) | 2/30 (7%) | 0/6 (0%) | 0/11 (0%) | 0/9 (0%) | 0/21 (0%) | 0/24 (0%) | 0/5 (0%) | | 0/4 (0%) | 14/32 (44%) | 0/11 (0%) | 0/0  (0%) |
| *Open Surgery* | 13/353  (4%) | 0/195 (0%) | 0/24 (0%) | 2/28 (7%) | 0/3 (0%) | 0/10 (0%) | 0/7 (0%) | 0/17 (0%) | 0/22 (0%) | 0/5 (0%) | | 0/4 (0%) | 11/27 (41%) | 0/11 (0%) | 0/0  (0%) |
| *MIS* | 16/409  (4%) | 0/270 (0%) | 0/18 (0%) | 2/25 (8%) | 0/4 (0%) | 0/8 (0%) | 0/9 (0%) | 0/19 (0%) | 0/20 (0%) | 0/3 (0%) | | 0/2 (0%) | 14/28 (50%) | 0/3 (0%) | 0/0  (0%) |
| **Transdermal opioids** | 10/466  (2%) | 2/283 (1%) | 1/30 (3%) | 0/30 (0%) | 1/6 (17%) | 1/11 (9%) | 1/9 (11%) | 1/21 (5%) | 0/24 (0%) | 0/5 (0%) | | 0/4 (0%) | 2/32 (6%) | 1/11 (9%) | 0/0  (0%) |
| *Open Surgery* | 8/353  (2%) | 2/195 (1%) | 0/24 (0%) | 0/28 (0%) | 1/3 (33%) | 0/10 (0%) | 1/7 (14%) | 1/17 (6%) | 0/22 (0%) | 0/5 (0%) | | 0/4 (0%) | 2/27 (7%) | 1/11 (9%) | 0/0  (0%) |
| *MIS* | 6/409  (1%) | 1/270 (0%) | 1/18 (6%) | 0/25 (0%) | 0/4 (0%) | 1/8 (13%) | 1/9 (11%) | 1/19 (5%) | 0/20 (0%) | 0/3 (0%) | | 0/2 (0%) | 1/28 (4%) | 0/3 (0%) | 0/0  (0%) |
| **Meperidine** | 1/466  (<1%) | 0/283 (0%) | 0/30 (0%) | 0/30 (0%) | 0/6 (0%) | 0/11 (0%) | 0/9 (0%) | 1/21 (5%) | 0/24 (0%) | 0/5 (0%) | | 0/4 (0%) | 0/32 (0%) | 0/11 (0%) | 0/0  (0%) |
| *Open Surgery* | 1/353 (<1%) | 0/195 (0%) | 0/24 (0%) | 0/28 (0%) | 0/3 (0%) | 0/10 (0%) | 0/7 (0%) | 1/17 (6%) | 0/22 (0%) | 0/5 (0%) | | 0/4 (0%) | 0/27 (0%) | 0/11 (0%) | 0/0  (0%) |
| *MIS* | 0/409  (0%) | 0/270 (0%) | 0/18 (0%) | 0/25 (0%) | 0/4 (0%) | 0/8 (0%) | 0/9 (0%) | 0/19 (0%) | 0/20 (0%) | 0/3 (0%) | | 0/2 (0%) | 0/28 (0%) | 0/3 (0%) | 0/0  (0%) |
| **Methadone** | 0/466  (0%) | 0/283 (0%) | 0/30 (0%) | 0/30 (0%) | 0/6 (0%) | 0/11 (0%) | 0/9 (0%) | 0/21 (0%) | 0/24 (0%) | 0/5 (0%) | | 0/4 (0%) | 0/32 (0%) | 0/11 (0%) | 0/0  (0%) |
| *Open Surgery* | 0/353  (0%) | 0/195 (0%) | 0/24 (0%) | 0/28 (0%) | 0/3 (0%) | 0/10 (0%) | 0/7 (0%) | 0/17 (0%) | 0/22 (0%) | 0/5 (0%) | | 0/4 (0%) | 0/27 (0%) | 0/11 (0%) | 0/0  (0%) |
| Table S6. Opioid medications prescribed at discharge in different world subregions (continued) | | | | | | | | | | | | | | | |
|  | Overall | Northern America | Latin America and the Caribbean | Northern Europe | Eastern Europe | Southern Europe | Western Europe | Eastern Asia | Western Asia | South-eastern Asia | | Southern Asia | Australia and New Zealand | Sub-Saharan Africa | Northern Africa |
| *MIS* | 0/409  (0%) | 0/270 (0%) | 0/18 (0%) | 0/25 (0%) | 0/4 (0%) | 0/8 (0%) | 0/9 (0%) | 0/19 (0%) | 0/20 (0%) | 0/3 (0%) | | 0/2 (0%) | 0/28 (0%) | 0/3 (0%) | 0/0  (0%) |
| **Nalbuphine** | 0/466  (0%) | 0/283 (0%) | 0/30 (0%) | 0/30 (0%) | 0/6 (0%) | 0/11 (0%) | 0/9 (0%) | 0/21 (0%) | 0/24 (0%) | 0/5 (0%) | | 0/4 (0%) | 0/32 (0%) | 0/11 (0%) | 0/0  (0%) |
| *Open Surgery* | 0/353  (0%) | 0/195 (0%) | 0/24 (0%) | 0/28 (0%) | 0/3 (0%) | 0/10 (0%) | 0/7 (0%) | 0/17 (0%) | 0/22 (0%) | 0/5 (0%) | | 0/4 (0%) | 0/27 (0%) | 0/11 (0%) | 0/0  (0%) |
| *MIS* | 0/409  (0%) | 0/270 (0%) | 0/18 (0%) | 0/25 (0%) | 0/4 (0%) | 0/8 (0%) | 0/9 (0%) | 0/19 (0%) | 0/20 (0%) | 0/3 (0%) | | 0/2 (0%) | 0/28 (0%) | 0/3 (0%) | 0/0  (0%) |

**Data are reported as frequency n (%).**

All the percentages were calculated based on the number of prescribers of each medication relative to the total number of opioid prescribers.

MIS: minimally invasive surgery.

| Table S7. Total Morphine Milligram Equivalents (MME) prescribed in different subregions (sensitivity analysis) | | |
| --- | --- | --- |
|  | **Open** | **Minimally invasive** |
|  | **MME [IQR]** | **MME [IQR]** |
| **All Regions** | 120 MMEs [75-180] | 100 MMEs [60-150] |
| **Americas** |  |  |
| Northern America | 120 MMEs [75-162.5] | 90 MMEs [60-150] |
| Latin America and the Caribbean | 50 MMEs [21.25-140] | 85.7 MMEs [30-145] |
| **Europe** |  |  |
| Northern Europe | 195 MMEs [126-329] | 175 MMEs [126-364] |
| Eastern Europe | 86.4 MMEs [5-90] | 30 MMEs [15-90] |
| Southern Europe | 126 MMEs [90-200] | 90 MMEs [84-140] |
| Western Europe | 140 MMEs [18-240] | 120 MMEs [37.5-150] |
| **Asia** |  |  |
| Eastern Asia | 100 MMEs [30-350] | 86.4 MMEs [30-280] |
| Western Asia | 40 MMEs [9-140] | 62.5 MMEs [25-140] |
| South-eastern Asia | 75 MMEs [75-75] | 45 MMEs [25-75] |
| Southern Asia | 87.5 MMEs [62.5-120] | 120 MMEs [100-140] |
| **Oceania** |  |  |
| Australia and New Zealand | 200 MMEs [80-560] | 205 MMEs [97.5-518.75] |
| **Africa** |  |  |
| Sub-Saharan Africa | 105 MMEs [50-140] | 100 MMEs [100-200] |
| Northern Africa | -- | -- |
| **Data are reported as median MME [IQR].**  For countries where opioid pills are pre-packed (i.e., the prescriber does not determine the number of pills received by patients at the pharmacy), this analysis considered that patients receive a standard box of 28 pills (6-day supply, ~4 pills per day). | | |

| Table S8. Non-opioid medications prescribed at discharge in different world subregions | | | | | | | | | | | | | | |
| --- | --- | --- | --- | --- | --- | --- | --- | --- | --- | --- | --- | --- | --- | --- |
|  | Overall | Northern America | Latin America and the Caribbean | Northern Europe | Eastern Europe | Southern Europe | Western Europe | Eastern Asia | Western Asia | South-eastern Asia | Southern Asia | Australia and New Zealand | Sub-Saharan Africa | Northern Africa |
| **Acetaminophen/Paracetamol, oral** | 545/817  (67%) | 251/308 (81%) | 60/115 (52%) | 38/44 (86%) | 15/56 (27%) | 52/58 (90%) | 9/17 (53%) | 19/96 (20%) | 42/52 (81%) | 5/7 (71%) | 6/7 (86%) | 31/32  (97%) | 12/18 (67%) | 5/7 (71%) |
| *Open Surgery (n=640)* | 417/640  (65%) | 167/211 (79%) | 44/89 (49%) | 35/41 (85%) | 10/44 (23%) | 46/51 (90%) | 6/11 (55%) | 16/80 (20%) | 39/47 (83%) | 5/7 (71%) | 6/7 (86%) | 26/27  (96%) | 12/18 (67%) | 5/7 (71%) |
| *MIS (n=760)* | 512/760  (67%) | 247/305 (81%) | 53/89 (60%) | 34/39 (87%) | 13/50 (26%) | 50/56 (89%) | 9/17 (53%) | 18/93 (19%) | 38/46 (83%) | 5/7 (71%) | 4/5 (80%) | 30/31  (97%) | 6/6 (100%) | 5/7 (71%) |
| **NSAIDs** | 454/817  (56%) | 167/308 (54%) | 70/115 (61%) | 19/44 (43%) | 38/56 (68%) | 31/58 (53%) | 4/17 (24%) | 47/96 (49%) | 35/52 (67%) | 7/7 (100%) | 4/7 (57%) | 14/32  (44%) | 12/18 (67%) | 6/7 (86%) |
| *Open Surgery (n=640)* | 356/640  (56%) | 121/211 (57%) | 60/89 (67%) | 15/41 (37%) | 29/44 (66%) | 25/51 (49%) | 1/11 (9%) | 37/80 (46%) | 31/47 (66%) | 7/7 (100%) | 4/7 (57%) | 8/27  (30%) | 12/18 (67%) | 6/7 (86%) |
| *MIS (n=760)* | 403/760  (53%) | 163/305 (53%) | 59/98 (60%) | 16/39 (41%) | 32/50 (64%) | 26/56 (46%) | 4/17 (24%) | 39/93 (42%) | 30/46 (65%) | 7/7 (100%) | 3/5 (60%) | 14/31  (45%) | 4/6 (67%) | 6/7 (86%) |
| **Dipyrone/Metamizole** | 78/817  (10%) | 2/308 (1%) | 35/115 (30%) | 0/44 (0%) | 9/56 (16%) | 21/58 (36%) | 7/17 (41%) | 0/96 (0%) | 3/52 (6%) | 0/7  (0%) | 0/7  (0%) | 0/32  (0%) | 1/18 (6%) | 0/7  (0%) |
| *Open Surgery (n=640)* | 60/640  (9%) | 2/211 (1%) | 24/89 (27%) | 0/41 (0%) | 7/44 (16%) | 18/51 (35%) | 5/11 (45%) | 0/80 (0%) | 3/47 (6%) | 0/7  (0%) | 0/7  (0%) | 0/27  (0%) | 1/18 (6%) | 0/7  (0%) |
| *MIS (n=760)* | 68/760  (9%) | 2/305 (1%) | 28/98 (29%) | 0/39 (0%) | 7/50 (14%) | 20/56 (36%) | 7/17 (41%) | 0/93 (0%) | 3/46 (7%) | 0/7  (0%) | 0/5  (0%) | 0/31  (0%) | 1/6 (17%) | 0/7  (0%) |
| **Gabapentinoids** | 67/817  (8%) | 58/308 (19%) | 1/115 (1%) | 3/44 (7%) | 1/56 (2%) | 0/58 (0%) | 0/17 (0%) | 0/96 (0%) | 2/52 (4%) | 0/7  (0%) | 0/7  (0%) | 2/32  (6%) | 0/18 (0%) | 0/7  (0%) |
| *Open Surgery (n=640)* | 45/640  (7%) | 38/211 (18%) | 1/89 (1%) | 3/41 (7%) | 0/44 (0%) | 0/51 (0%) | 0/11 (0%) | 0/80 (0%) | 1/47 (2%) | 0/7  (0%) | 0/7  (0%) | 2/27  (7%) | 0/18 (0%) | 0/7  (0%) |
| **Table S8. Non-opioid medications prescribed at discharge in different world subregions (continued)** | | | | | | | | | | | | | | |
|  | Overall | Northern America | Latin America and the Caribbean | Northern Europe | Eastern Europe | Southern Europe | Western Europe | Eastern Asia | Western Asia | South-eastern Asia | Southern Asia | Australia and New Zealand | Sub-Saharan Africa | Northern Africa |
| *MIS (n=760)* | 61/760  (8%) | 55/305 (18%) | 0/98 (0%) | 1/39 (3%) | 1/50 (2%) | 0/56 (0%) | 0/17 (0%) | 0/93 (0%) | 2/46 (4%) | 0/7  (0%) | 0/5  (0%) | 2/31  (6%) | 0/6  (0%) | 0/7  (0%) |
| **Nefopam** | 1/817  (<1%) | 0/308 (0%) | 0/115 (0%) | 0/44 (0%) | 0/56 (0%) | 0/58 (0%) | 1/17 (6%) | 0/96 (0%) | 0/52 (0%) | 0/7  (0%) | 0/7  (0%) | 0/32  (0%) | 0/18 (0%) | 0/7  (0%) |
| *Open Surgery (n=640)* | 1/640  (<1%) | 0/211 (0%) | 0/89 (0%) | 0/41 (0%) | 0/44 (0%) | 0/51 (0%) | 1/11 (9%) | 0/80 (0%) | 0/47 (0%) | 0/7  (0%) | 0/7  (0%) | 0/27  (0%) | 0/18 (0%) | 0/7  (0%) |
| *MIS (n=760)* | 1/760  (<1%) | 0/305 (0%) | 0/98 (0%) | 0/39 (0%) | 0/50 (0%) | 0/56 (0%) | 1/17 (6%) | 0/93 (0%) | 0/46 (0%) | 0/7  (0%) | 0/5  (0%) | 0/31  (0%) | 0/6  (0%) | 0/7  (0%) |

**Data are reported as frequency n (%) or median (IQR).**The total numbers of participants (n=817) is less than the cumulative frequency of open surgery (n=640) and MIS (n=760) because many participants conducted both types of procedures.
NSAIDs: non-steroidal anti-inflammatory drugs, MIS: minimally invasive surgery.

|  |
| --- |

| Table S9. NSAIDs medications prescribed at discharge in different world subregions | | | | | | | | | | | | | | |
| --- | --- | --- | --- | --- | --- | --- | --- | --- | --- | --- | --- | --- | --- | --- |
|  | Overall | Northern America | Latin America and the Caribbean | Northern Europe | Eastern Europe | Southern Europe | Western Europe | Eastern Asia | Western Asia | South-eastern Asia | Southern Asia | Australia and New Zealand | Sub-Saharan Africa | Northern Africa |
| **Ibuprofen** | 225/454 (50%) | 115/167 (69%) | 17/70 (24%) | 14/19 (74%) | 12/38 (32%) | 21/31 (68%) | 3/4 (75%) | 13/47 (28%) | 17/35 (49%) | 0/7  (0%) | 1/4 (25%) | 8/14 (57%) | 4/12 (33%) | 0/6 (0%) |
| *Open Surgery (n=660)* | 183/356 (51%) | 88/121 (73%) | 16/60 (27%) | 11/15 (73%) | 11/29 (38%) | 17/25 (68%) | 1/1 (100%) | 12/37 (32%) | 17/31 (55%) | 0/7  (0%) | 1/4 (25%) | 5/8 (63%) | 4/12 (33%) | 0/6 (0%) |
| *MIS (n=760)* | 220/403 (55%) | 114/163 (70%) | 13/59 (22%) | 10/16 (63%) | 8/32 (25%) | 19/26 (73%) | 3/4 (75%) | 10/39 (26%) | 15/30 (50%) | 0/7  (0%) | 0/3 (0%) | 8/14 (57%) | 2/4 (50%) | 0/6 (0%) |
| **Ketorolac** | 103/454 (23%) | 11/167 (7%) | 40/70 (57%) | 4/19 (21%) | 27/38 (71%) | 8/31 (26%) | 0/4 (0%) | 6/47 (13%) | 4/35 (11%) | 0/7  (0%) | 2/4 (50%) | 0/14 (0%) | 0/12 (0%) | 1/6 (17%) |
| *Open Surgery (n=640)* | 85/356 (24%) | 9/121 (7%) | 36/60 (60%) | 3/15 (20%) | 20/29 (69%) | 8/25 (32%) | 0/1 (0%) | 4/37 (11%) | 2/31 (6%) | 0/7  (0%) | 2/4 (50%) | 0/8 (0%) | 0/12 (0%) | 1/6 (17%) |
| *MIS (n=760)* | 88/403 (22%) | 11/163 (7%) | 36/59 (61%) | 3/16 (19%) | 22/32 (69%) | 6/26 (23%) | 0/4 (0%) | 5/39 (13%) | 3/30 (10%) | 0/7  (0%) | 1/3 (33%) | 0/14 (0%) | 0/4 (0%) | 1/6 (17%) |
| **Celecoxib** | 96/454 (21%) | 38/167 (23%) | 8/70 (11%) | 3/19 (16%) | 1/38 (3%) | 3/31 (10%) | 1/4 (25%) | 19/47 (40%) | 8/35 (23%) | 7/7  (100%) | 0/4 (0%) | 6/14 (43%) | 0/12 (0%) | 2/6 (33%) |
| *Open Surgery (n=640)* | 70/356 (20%) | 25/121 (21%) | 7/60 (12%) | 2/15 (13%) | 1/29 (3%) | 2/25 (8%) | 0/1 (0%) | 15/37 (41%) | 6/31 (19%) | 7/7  (100%) | 0/4 (0%) | 3/8 (38%) | 0/12 (0%) | 2/6 (33%) |
| *MIS (n=760)* | 88/403 (22%) | 35/163 (21%) | 8/59 (14%) | 3/16 (19%) | 1/32 (3%) | 1/26 (4%) | 1/4 (25%) | 16/39 (41%) | 8/30 (27%) | 7/7  (100%) | 0/3 (0%) | 6/14 (43%) | 0/4 (0%) | 2/6 (33%) |
| **Diclofenac** | 82/454 (18%) | 3/167 (2%) | 19/70 (27%) | 3/19 (16%) | 7/38 (18%) | 3/31 (10%) | 0/4 (0%) | 11/47 (23%) | 19/35 (54%) | 0/7  (0%) | 4/4 (100%) | 0/14 (0%) | 9/12 (75%) | 4/6 (67%) |
| *Open Surgery (n=640)* | 75/356 (21%) | 3/121 (2%) | 15/60 (25%) | 3/15 (20%) | 6/29 (21%) | 3/25 (12%) | 0/1 (0%) | 10/37 (27%) | 18/31 (58%) | 0/7  (0%) | 4/4 (100%) | 0/8 (0%) | 9/12 (75%) | 4/6 (67%) |
| Table S9. NSAIDs medications prescribed at discharge in different world subregions (continued) | | | | | | | | | | | | | | |
|  | Overall | Northern America | Latin America and the Caribbean | Northern Europe | Eastern Europe | Southern Europe | Western Europe | Eastern Asia | Western Asia | South-eastern Asia | Southern Asia | Australia and New Zealand | Sub-Saharan Africa | Northern Africa |
| *MIS (n=760)* | 56/403  (14%) | 2/163 (1%) | 14/59 (24%) | 2/16 (13%) | 5/32 (16%) | 3/26 (12%) | 0/4 (0%) | 7/39 (18%) | 15/30 (50%) | 0/7  (0%) | 3/3 (100%) | 0/14 (0%) | 1/4 (25%) | 4/6 (67%) |
| **Naproxen** | 39/454 (9%) | 28/167 (17%) | 1/70 (1%) | 1/19 (5%) | 2/38 (5%) | 1/31 (3%) | 0/4 (0%) | 2/47 (4%) | 3/35 (9%) | 0/7  (0%) | 0/4 (0%) | 0/14 (0%) | 1/12 (8%) | 0/6 (0%) |
| *Open Surgery (n=640)* | 27/356 (8%) | 18/121 (15%) | 0/60 (0%) | 1/15 (7%) | 2/29 (7%) | 1/25 (4%) | 0/1 (0%) | 2/37 (5%) | 2/31 (6%) | 0/7  (0%) | 0/4 (0%) | 0/8 (0%) | 1/12 (8%) | 0/6 (0%) |
| *MIS (n=760)* | 32/403 (8%) | 25/163 (15%) | 1/59 (2%) | 0/16 (0%) | 1/32 (3%) | 0/26 (0%) | 0/4 (0%) | 1/39 (3%) | 3/30 (10% | 0/7  (0%) | 0/3 (0%) | 0/14 (0%) | 1/4 (25%) | 0/6 (0%) |

**Data are reported as frequency n (%).**All the percentages were calculated based on the number of prescribers of each medication relative to the total number of NSAIDs prescribers.

NSAIDs: non-steroidal anti-inflammatory drugs, MIS: minimally invasive surgery.

# Table S10. Bayesian model averaging (BMA) analysis of potential predictors opioid prescribing at discharge after minimally invasive surgery (Multiple BMA analysis) (n=760)

| **Potential predictors** | **OR [95% CI]** | **PEP** |
| --- | --- | --- |
| **Subregion of practice** |  | 100 |
| Northern America | Reference |  |
| Australia and New Zealand | 1.20 [0.34-4.17] |  |
| Latin America and the Caribbean | 0.03 [0.02-0.05] |  |
| Western Asia | 0.10 [0.05-0.20] |  |
| South-eastern Asia | 0.10 [0.02-0.47] |  |
| Southern Asia | 0.09 [0.01-0.54] |  |
| Eastern Asia | 0.03 [0.02-0.06] |  |
| Northern Europe | 0.23 [0.11-0.48] |  |
| Eastern Europe | 0.01 [0.00-0.03] |  |
| Western Europe | 0.14 [0.05-0.40] |  |
| Southern Europe | 0.02 [0.01-0.05] |  |
| Sub-Saharan Africa | 0.13 [0.03-0.67] |  |
| Northern Africa^a^ | 0% opioid prescription rate |  |
| **Years of independent practice** |  | 0 |
| 0 | Reference |  |
| 1-6 | 1 |  |
| 6-10 | 1 |  |
| 11-20 | 1 |  |
| >20 | 1 |  |
| **Practice setting** |  | 0 |
| Academic | Reference |  |
| Community or other | 1 |  |
| **Average length of stay, days** | 1 | 0 |
| **Procedures performed annually** |  | 0 |
| <11 | Reference |  |
| 11-30 | 1 |  |
| 31-60 | 1 |  |
| 61-100 | 1 |  |
| >100 | 1 |  |
| **NSAIDs prescription at discharge (Yes)** | 0.95 [0.71-1.27] | 14 |
| In Bayesian model averaging, ORs are calculated from the mean of the beta coefficients (weighted averages) of all possible models and PEP (reported in %) reflects how likely it is for specific variables to predict the outcome of interest.  ORs should be interpreted the between-group difference in odds of opioid prescribing (for nominal/dichotomous predictors) or difference in odds for every 1-unit change (for continuous predictors), assuming all other variables are held constant. When the OR is exactly 1, it means there is no association between the predictor and the outcome (PEP=0).  ^a^ Variables indicating perfect prediction were omitted from the model.  OR: odds ratio; PEP: posterior effect probability; NSAIDs: non-steroidal anti-inflammatory drugs, CI: Confidence Interval. | | |

# Table S11. Bayesian model averaging (BMA) analysis of potential predictors of opioid prescribing at discharge after open surgery (Multiple BMA analysis) (n=640)

| **Potential predictors** | **OR [95% CI]** | **PEP** |
| --- | --- | --- |
| **Subregion of practice** |  | 100 |
| North America | Reference |  |
| Latin America and the Caribbean | 0.03 [0.02-0.06] |  |
| Western Asia | 0.07 [0.03-0.16] |  |
| South-eastern Asia | 0.21 [0.04-1.17] |  |
| Southern Asia | 0.11 [0.02-0.54] |  |
| Eastern Asia | 0.02 [0.01-0.05] |  |
| Northern Europe | 0.18 [0.08-0.41] |  |
| Eastern Europe | 0.01 [0.00-0.02] |  |
| Western Europe | 0.14 [0.04-0.54] |  |
| Southern Europe | 0.02 [0.01-0.05] |  |
| Sub-Saharan Africa | 0.13 [0.04-0.38] |  |
| Australia and New Zealand^a^ | 0% opioid prescription rate |  |
| Northern Africa^a^ | 100% opioid prescription rate |  |
| **Years of independent practice** |  | 0 |
| 0 | Reference |  |
| 1-6 | 1 |  |
| 6-10 | 1 |  |
| 11-20 | 1 |  |
| >20 | 1 |  |
| **Practice setting** |  | 6 |
| Academic | Reference |  |
| Community or other | 0.98 [0.83-1.17] |  |
| **Average length of stay, days** | 1.00 [0.97-1.03] | 4 |
| **Procedures performed annually** |  | 0 |
| <11 | Reference |  |
| 11-30 | 1 |  |
| 31-60 | 1 |  |
| 61-100 | 1 |  |
| >100 | 1 |  |
| **NSAIDs prescription at discharge (Yes)** | 0.95 [0.69-1.30] | 14 |
| In Bayesian model averaging, ORs are calculated from the mean of the beta coefficients (weighted averages) of all possible models and PEP (reported in %) reflects how likely it is for specific variables to predict the outcome of interest.  ORs should be interpreted the between-group difference in odds of opioid prescribing (for nominal/dichotomous predictors) or difference in odds for every 1-unit change (for continuous predictors), assuming all other variables are held constant. When the OR is exactly 1, it means there is no association between the predictor and the outcome (PEP=0).  ^a^ Variables indicating perfect prediction were omitted from the model.  OR: odds ratio; PEP: posterior effect probability; NSAIDs: non-steroidal anti-inflammatory drugs, CI: Confidence Interval. | | |
